# Supplementary material for: A hydrophobic funnel governs monovalent cation selectivity in the ion channel TRPM5
Source: Biophys J. 2024 Jul 30;123(19):3304–16. doi: 10.1016/j.bpj.2024.07.035 (PMC11480762; doi:10.1016/j.bpj.2024.07.035)
Supplement: Document S1. Figures S1–S5 and Tables S1–S3 [file mmc1.pdf]

Biophysical Journal, Volume *123*

Supplemental information

A hydrophobic funnel governs monovalent cation selectivity in the ion channel TRPM5

Callum M. Ives, Alp Tegin Şahin, Neil J. Thomson, and Ulrich Zachariae

Supplementary Information

Summary of MD simulations used within this study

Table S1. Summary of CompEL simulation details of the TRPM5 channel. All simulations were conducted in a di-cationic solution of 75 mM NaCl and 75 mM $CaCl_2$. In all simulations, the Ca^{2+} cations occupying the Ca_{TMD} were modelled, and remained bound for the duration of the simulations.

**Protein**	TRPM5				
**Structure**	7MBS<br>(698-1020)				
**Force field**	CHARMM36m				
**Water**	TIP3P				
In silico electrophysiology methodology	CompEL<br>(anti-parallel with a 9:1 concentration gradient)				
**Ion**	75 mM NaCl + 75mm $CaCl_2$<br>266 Na^+ (CHARMM36m)<br>274 Ca^{2+} (Zhang et al.)<br>814 Cl^- (CHARMM36m)				
**Independent simulations**	3	3	3	3	3
**Total simulation time (μs)**	1.5	1.5	1.5	1.5	0.45
**Total aggregated simulation time (μs)**	3	3	3	3	0.9
**Ionic ratios between compartments (Extracellular : Intracellular)**	239 : 27 Na^+<br>239 : 35 Ca^{2+}<br>681 : 133 Cl^-	239 : 27 Na^+<br>239 : 35 Ca^{2+}<br>680 : 134 Cl^-	239 : 27 Na^+<br>239 : 35 Ca^{2+}<br>677 : 137 Cl^-	239 : 27 Na^+<br>239 : 35 Ca^{2+}<br>673 : 141 Cl^-	239 : 27 Na^+<br>239 : 35 Ca^{2+}<br>684 : 130 Cl^-
**Estimated voltage (mV)**	-50	-130	-380	-610	0
**Permeation events**	15 Na^+<br>0 Ca^{2+}<br>0 Cl^-	18 Na^+<br>0 Ca^{2+}<br>0 Cl^-	32 Na^+<br>19 Ca^{2+}<br>1 Cl^-	115 Na^+<br>168 Ca^{2+}<br>6 Cl^-	0 Na^+<br>0 Ca^{2+}<br>0 Cl^-
**Total number of permeation events**	15	18	52	289	0

Table S2. Summary of external applied field simulation details of the TRPM5 channel. All simulations were conducted in a mono-cationic solution of either 150 mM NaCl, 150 mM KCl, or 150 mM $CaCl_2$. In all simulations, the Ca^{2+} cations occupying the Ca_{TMD} were modelled, and remained bound for the duration of the simulations.

**Protein**	TRPM5		
**Structure**	7MBS<br>(698-1020)		
**Force field**	CHARMM36m		
**Water**	TIP3P		
In silico electrophysiology methodology	External applied field		
**Ion**	150 mM NaCl<br>267 Na^+ (CHARMM36m)<br>275 Cl^- (CHARMM36m)<br>4 Ca^{2+} (CHARMM36m)	150 mM KCl<br>267 K+ (CHARMM36m)<br>275 Cl^- (CHARMM36m)<br>4 Ca^{2+} (CHARMM36m)	150 mM $CaCl_2$<br>271 Ca^{2+} (Zhang *et al.*)<br>542 Cl^- (CHARMM36m)
**Independent simulations**	3	3	3
**Total simulation time (μs)**	0.75	0.75	0.75
**Estimated voltage (mV)**	-340	-340	-340
**Permeation events**	83 Na^+<br>0 Cl^-	34 K+<br>0 Cl^-	54 Ca^{2+}<br>0 Cl^-
**Total number of permeation events**	83	34	54

**Protein**	TRPM5			
**Structure**	7MBS<br>(698-1020)			
**Force field**	CHARMM36m			
**Water**	TIP3P			
In silico electrophysiology methodology	External applied field			
**Ion**	150 mM NaCl<br>267 Na^+ (CHARMM36m)<br>275 Cl^- (CHARMM36m)<br>4 Ca^{2+} (CHARMM36m)	150 mM $CaCl_2$<br>271 Ca^{2+} (Zhang et al.)<br>542 Cl^- (CHARMM36m)	150 mM NaCl<br>267 Na^+ (CHARMM36m)<br>275 Cl^- (CHARMM36m)<br>4 Ca^{2+} (CHARMM36m)	150 mM $CaCl_2$<br>271 Ca^{2+} (Zhang et al.)<br>542 Cl^- (CHARMM36m)
**Independent simulations**	3	3	3	3
**Total simulation time (μs)**	0.75	0.75	0.75	0.75
**Estimated voltage (mV)**	-50	-50	-200	-200
**Permeation events**	4 Na^+<br>0 Cl^-	0 Ca^{2+}<br>0 Cl^-	15 Na^+<br>0 Cl^-	4 Ca^{2+}<br>0 Cl^-
**Total number of permeation events**	4	0	15	4

Table S3. Summary of external applied field simulation details of the TRPM5 F904T channel. All simulations were conducted in a mono-cationic solution of either 150 mM NaCl, or 150 mM $CaCl_2$. In all simulations, the Ca^{2+} cations occupying the Ca_{TMD} were modelled, and remained bound for the duration of the simulations.

**Protein**	TRPM5 F904T			
**Structure**	7MBS<br>(698-1020)			
**Force field**	CHARMM36m			
**Water**	TIP3P			
**In silico electrophysiology methodology**	External applied field			
**Ion**	150 mM NaCl<br>267 Na+ (CHARMM36m)<br>275 Cl- (CHARMM36m)<br>4 Ca2+ (CHARMM36m)	150 mM CaCl2<br>271 Ca2+ (Zhang et al.)<br>542 Cl- (CHARMM36m)	150 mM NaCl<br>267 Na+ (CHARMM36m)<br>275 Cl- (CHARMM36m)<br>4 Ca2+ (CHARMM36m)	150 mM CaCl2<br>271 Ca2+ (Zhang et al.)<br>542 Cl- (CHARMM36m)
**Independent simulations**	3	3	3	3
**Total simulation time (μs)**	0.75	0.75	0.75	0.75
**Estimated voltage (mV)**	-130	-130	-200	-200
**Permeation events**	27 Na+<br>0 Cl-	13 Ca2+<br>0 Cl-	88 Na+<br>0 Cl-	28 Ca2+<br>0 Cl-
**Total number of permeation events**	27	13	88	28

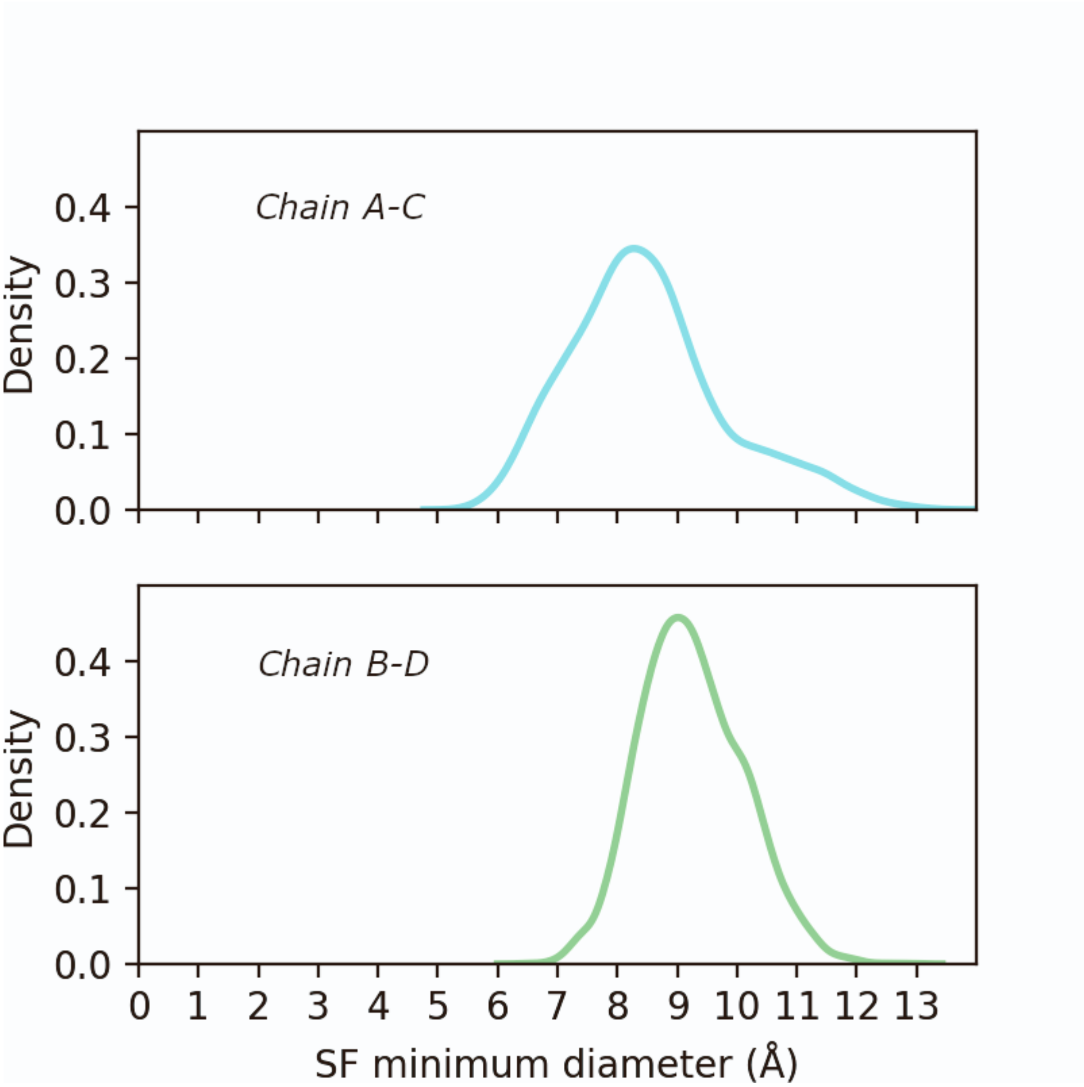

Figure S1. Minimum diameter of the TRPM5 selectivity filter measured at the SF constriction formed by residues G905 (carbonyl groups). The top panel shows the distance between the residues of chains A and C, the lower panel between chains B and D.

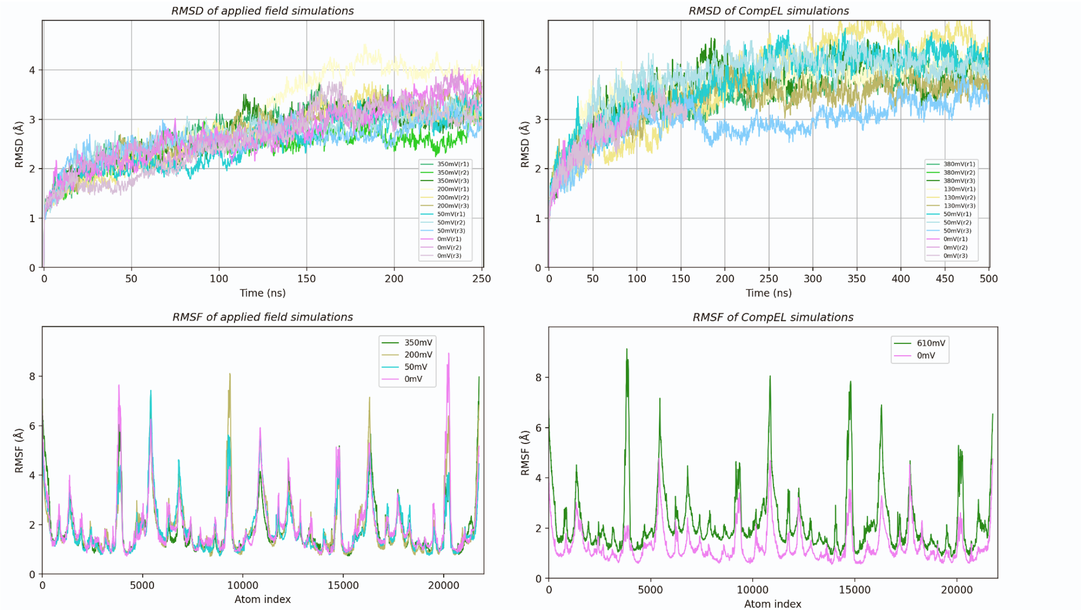

Figure S2. Root-mean-square deviation (RMSD) and root-mean-square fluctuation (RMSF) of the channel in the simulations. Note that the CompEL simulations at 0 mV are shorter than those at a higher voltage.

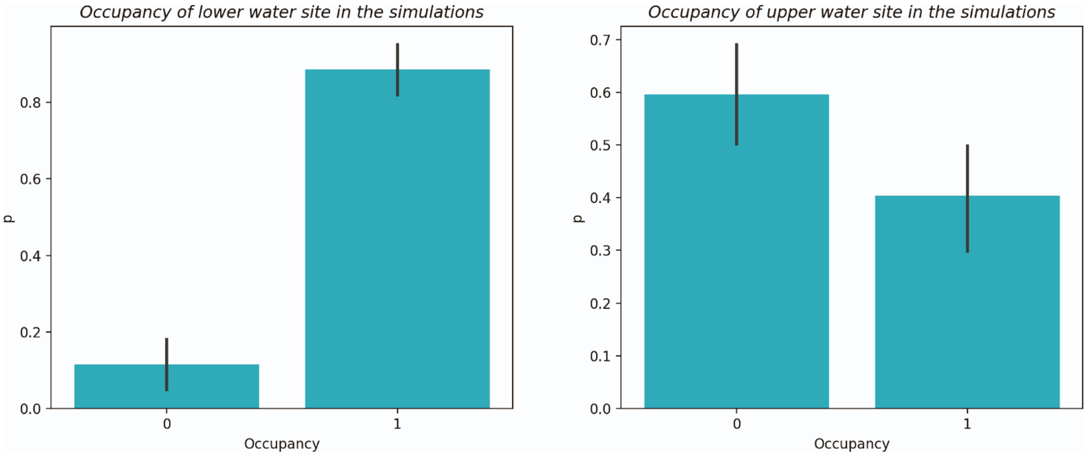

Figure S3. Occupancy of the water sites near Q906 in the SF identified in the cryo-EM structures.

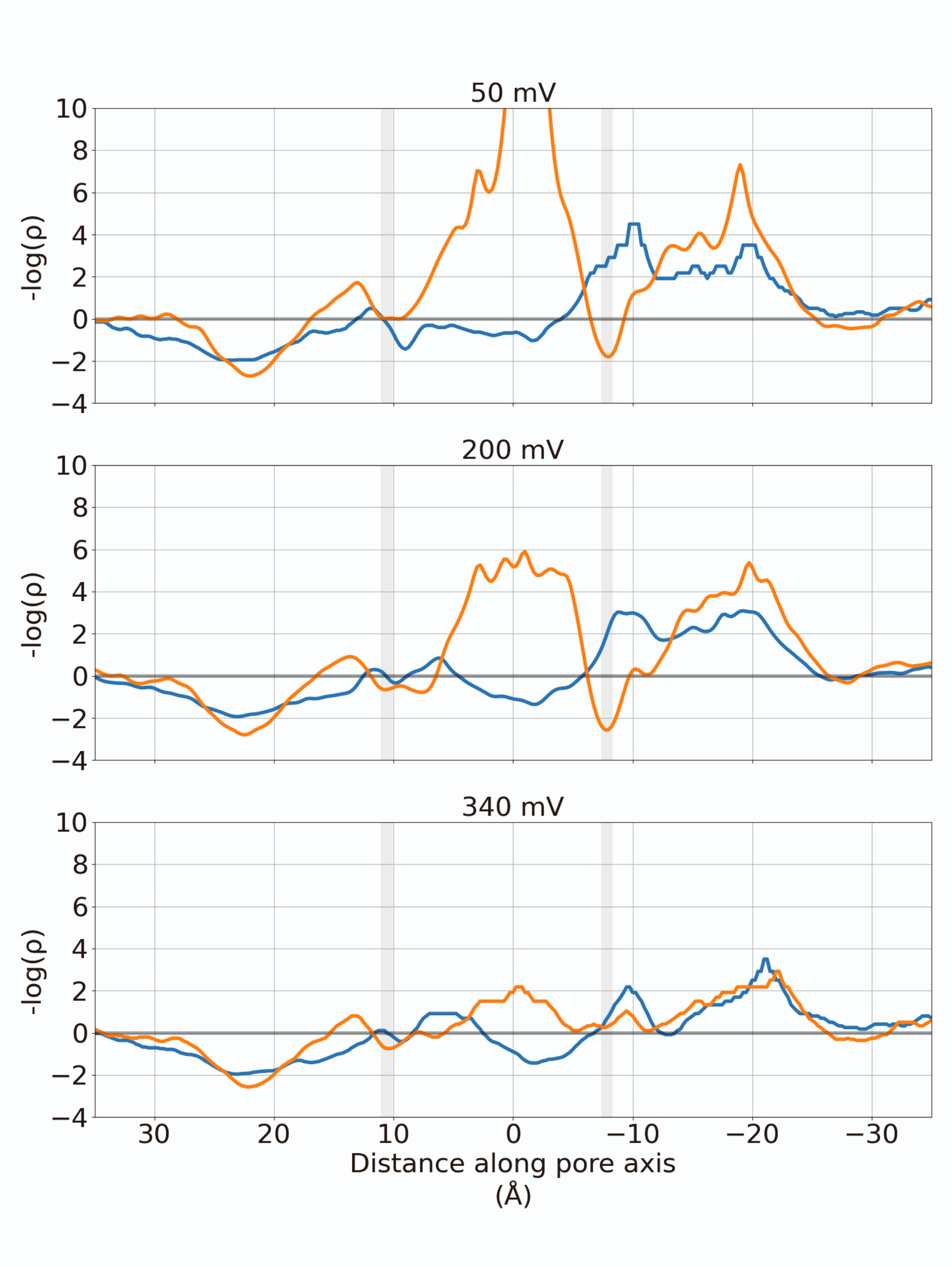

Figure S4. Negative logarithmic density profiles of permeating cations along the pore of the TRPM5 at different voltages. These simulations were performed in a mono-cationic solution, with an external applied electric field used to produce transmembrane voltages of ~ -50 mV (*top*), ~ -200 mV (*centre*), and ~ -340 mV (*bottom*). The logarithmic ion densities represent quasi-free energies (with a nominal unit of kT). The location of the pore constrictions formed by Q906 (*upper*) and I966 (*lower*) are represented as gray regions. Both plots have been smoothed using a Gaussian filter with a sigma value of 2.

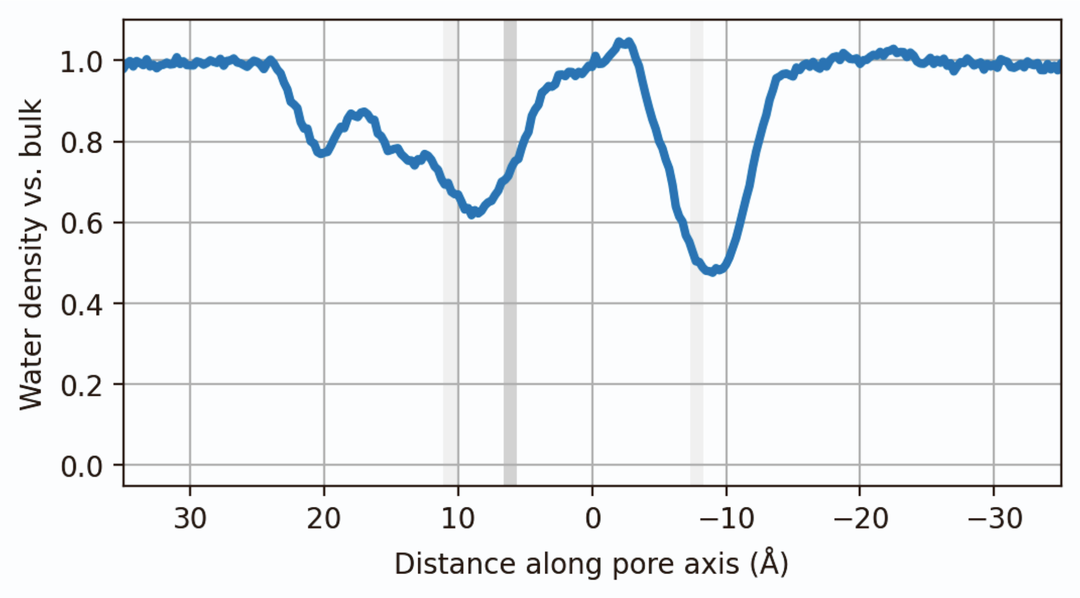

Figure S5. Water density along the pore axis of TRPM5 compared to bulk solution. Functionally important residue locations in the pore are shown as gray bars.
