# Supplementary material for: A hydrophobic funnel governs monovalent cation selectivity in the ion channel TRPM5
Source: Biophys J. 2024 Jul 30;123(19):3304–16. doi: 10.1016/j.bpj.2024.07.035 (PMC11480762; doi:10.1016/j.bpj.2024.07.035)
Supplement: Document S2. Article plus supporting material [file mmc2.pdf]

Biophysical Journal
Article

Biophysical Society

A hydrophobic funnel governs monovalent cation selectivity in the ion channel TRPM5

Callum M. Ives,[1] Alp Tegin Şahin,[1,2] Neil J. Thomson,[1] and Ulrich Zachariae[1,3,*]
[1]Computational Biology, School of Life Sciences, University of Dundee, Dundee, United Kingdom; [2]School of Medicine, University of St Andrews, St Andrews, United Kingdom; and [3]Biological Chemistry and Drug Discovery, School of Life Sciences, University of Dundee, Dundee, United Kingdom

ABSTRACT A key capability of ion channels is the facilitation of selective permeation of certain ionic species across cellular membranes at high rates. Due to their physiological significance, ion channels are of great pharmaceutical interest as drug targets. The polymodal signal-detecting transient receptor potential (TRP) superfamily of ion channels forms a particularly promising group of drug targets. While most members of this family permeate a broad range of cations including Ca^{2+}, TRPM4 and TRPM5 are unique due to their strong monovalent selectivity and impermeability for divalent cations. Here, we investigated the mechanistic basis for their unique monovalent selectivity by in silico electrophysiology simulations of TRPM5. Our simulations reveal an unusual mechanism of cation selectivity, which is underpinned by the function of the central channel cavity alongside the selectivity filter. Our results suggest that a subtle hydrophobic barrier at the cavity entrance ("hydrophobic funnel") enables monovalent but not divalent cations to pass and occupy the cavity at physiologically relevant membrane voltages. Monovalent cations then permeate efficiently by a cooperative, distant knock-on mechanism between two binding regions in the extracellular pore vestibule and the central cavity. By contrast, divalent cations do not enter or interact favorably with the channel cavity due to its raised hydrophobicity. Hydrophilic mutations in the transition zone between the selectivity filter and the central channel cavity abolish the barrier for divalent cations, enabling both monovalent and divalent cations to traverse TRPM5.

SIGNIFICANCE TRPM5 is a channel protein in the transient receptor potential (TRP) channel family, whose members play key roles in the physiology underlying human senses, including the sensation of temperature and pain. TRPM5 is an essential element in the perception of taste in humans, but has also been implicated in insulin secretion, making it a potential drug target for diabetes treatments. Unlike other TRP channels, TRPM5 has been shown to be strongly selective for monovalent cations versus divalent cations. In this work, we used molecular dynamics simulations under voltage and ion gradients to investigate ion permeation across TRPM5, which revealed a new cation selectivity mechanism underpinned by the function of a "hydrophobic funnel" at the internal exit of the selectivity filter.

INTRODUCTION

The translocation of ions across cellular and organellar membranes via ion channels is essential to ensure cellular ionic homeostasis and provides a key pathway of intra- and intercellular communication. Ion channels catalyze the permeation of ions across the membrane up to an order of 10^8 ions per second, while at the same time often displaying strict selectivity for particular ionic species (1–3). The transient receptor potential (TRP) superfamily of ion channels comprises a large group of cation-selective channels that are implicated in a wide range of physiological processes, including the sensation of temperature and pain (4–6). Due to their physiological importance, TRP channels are associated with a large number of pathological conditions (7), including in the etiology of several rare, genetic conditions. Many members of the TRP channel superfamily constitute major pharmaceutical target proteins (8,9).

Within the TRP channel superfamily, TRPM (transient receptor potential melastatin) channels form the largest subfamily, consisting of eight members (TRPM1-8) (10,11). TRPM channels assemble as homotetramers, in which each subunit provides six transmembrane helices (S1-S6),

Submitted November 16, 2023, and accepted for publication July 25, 2024.
*Correspondence: u.zachariae@dundee.ac.uk
Callum M. Ives's present address is Department of Chemistry and Hamilton Institute, Maynooth University, Maynooth, Co. Kildare, Ireland.
Callum M. Ives and Alp Tegin Şahin contributed equally to this work.
Editor: Philip Biggin.
https://doi.org/10.1016/j.bpj.2024.07.035
© 2024 The Author(s). Published by Elsevier Inc. on behalf of Biophysical Society.
This is an open access article under the CC BY license (http://creativecommons.org/licenses/by/4.0/).

a cytosolic N-terminal domain composed of four melastatin homology regions, and a cytosolic C-terminal coiled-coil domain (12,13). They also exhibit large regions of intrinsic disorder (14). In keeping with most members of the TRP superfamily, TRPM channels are described as being cation nonselective, that is, they conduct cations but do not differentiate substantially between cationic species. However, in the TRPM subfamily, TRPM4 and TRPM5 are exceptions to this observation, since both channels are selective for monovalent cations and impermeable to divalent cations (15,16). Thereby, TRPM4 and TRPM5 are the only members of the wider TRP superfamily to display selectivity for monovalent cations.

Although TRPM4 and TRPM5 are close homologs, sharing both a high degree of sequence homology and similar biophysical characteristics, there are some variations in their activation mechanisms. For example, while both channels are activated by raised intracellular Ca^{2+} concentrations, TRPM5 is approximately 20-fold more sensitive to Ca^{2+} than TRPM4 (17). Ion conduction through TRPM5 has been implicated in the sensation of sweet, bitter, and umami tastes in type II taste bud cells (18–20), and in the secretion of insulin by pancreatic β cells (21,22). Consequently, TRPM5 is a potential drug target for a number of conditions, including metabolic conditions such as type II diabetes mellitus (23). Several molecular structures of the TRPM4 and TRPM5 channels have been published to date; however, open-state structures have only been solved for TRPM5 (24) (PDB: 7MBS and 7MBQ).

In this work, we set out to characterize the cation permeation mechanism of the TRPM5 channel, focusing in particular on the basis for its monovalent cation selectivity, by conducting atomistic molecular dynamics (MD) simulations and in silico electrophysiology of the open-state structure of *Danio rerio* TRPM5 (24) (PDB: 7MBS) in solutions of Na^+, K^+, and Ca^{2+} ions. We recorded more than 700 individual ion permeation events from over 20 μs of aggregated time in our in silico electrophysiology simulations.

Our findings reveal a new mechanism of ion selectivity, based on a hydrophobic barrier at the entrance to the central channel cavity, which shields the cavity from an influx of divalent cations. In this way, the central cavity forms a binding site for monovalent cations, but not for divalent cations. The conduction of monovalent ions thus becomes a synergistic process incorporating cooperativity between multiple binding sites.

MATERIALS AND METHODS

TRPM5 system construction

A truncated TRPM5 simulation system consisting of the membrane domain of the channel was constructed by using residues 698–1020, including the resolved *N*-acetyl-β-D-glucosamine of the glycosylated N921 residue, of the *Danio rerio* TRPM5 structure (24) (PDB: 7MBS). We also modeled the bound Ca^{2+} cations occupying the Ca_{TMD} binding sites at E768 and D797 in each subunit, which have been proposed to be implicated in Ca^{2+}-dependent activation of TRPM5. The system was built using the CHARMM-GUI server (25). The charged N- and C-terminal residues were neutralized by capping with acetyl (ACE) and *N*-methylamide (CT3) groups, respectively. All missing nonterminal residues were modeled using CHARMM-GUI (26).

The structure was aligned in the membrane using the PPM server (27), inserted into a 1-palmitoyl-2-oleoyl-*sn*-glycerol-3-phosphocholine (POPC) bilayer of 160 × 160 Å size with the CHARMM-GUI membrane builder (28,29), and then solvated. Ions were added with GROMACS 2020.2 (30,31) to neutralize any system charges and ions added to a concentration of either 150 mM NaCl, 150 mM KCl, and 150 mM $CaCl_2$ (referred to as monocationic solutions) or a mixture of 75 mM NaCl and 75 mM $CaCl_2$ (referred to as dicationic solutions). In the case of simulations containing Ca^{2+}, the standard CHARMM36m parameters for Ca^{2+} ions were then replaced with the multisite Ca^{2+} of Zhang et al. (32). This multisite model has been used to investigate Ca^{2+} permeation in a number of channels, including the type 1 ryanodine receptor (32,33), AMPA receptors (34), the E protein of SARS-CoV-2 (35), and TRPV channels (36,37). The CHARMM36m parameters were retained for the immobile Ca^{2+} ions in the Ca_{TMD} sites.

MD simulation details

All simulations were performed using GROMACS 2020.2 (30,31) or GROMACS 2022 (38), together with the CHARMM36m force field for the protein, lipids, and ions (except for Ca^{2+} as noted above) (39). The TIP3P water model was used to model solvent molecules (40). The system was energy minimized and equilibrated using the suggested equilibration input scripts from CHARMM-GUI (41). In brief, the system was equilibrated using the NPT ensemble for a total time of 1.85 ns with the force constraints on the system components being gradually released over six equilibration steps. The systems were then further equilibrated by performing a 15-ns simulation with no electric field applied. To prevent closing of the lower hydrophobic gate of the pore, harmonic restraints were applied to maintain the distance between the α-carbon atoms of the lower gate residue I966 of each respective chain. The temperature was maintained at T = 310 K using the Nosé-Hoover thermostat (42), and the pressure was maintained semiisotropically at 1 bar using the Parrinello-Rahman barostat (43). Periodic boundary conditions were used throughout the simulations. Long-range electrostatic interactions were modeled using the particle-mesh Ewald method (44) with a cutoff of 12 Å. The LINCS algorithm (45) was used to constrain bond lengths involving bonds with hydrogen atoms. Hydrogen mass repartitioning of the system was used to allow the use of 4-fs integration time steps in simulations of NaCl solutions. The multisite Ca^{2+} model used for simulations of $CaCl_2$, however, is incompatible with a 4-fs time step, and therefore any simulations involving Ca^{2+} cations were performed with hydrogen mass repartitioning but at a time step of 2 fs. A summary of all simulations performed is presented in Table 1, and in more detail in Tables S1 and S2.

CompEL simulations

We employed the computational electrophysiology (CompEL) protocol (46,47) of GROMACS to create a transmembrane voltage and drive ion permeation in an antiparallel double-membrane system, such that both channels experienced the same voltage polarity with negative polarity in the intracellular region. Simulations were performed in a dicationic solution of 75 mM NaCl and 75 mM $CaCl_2$ with a range of ionic imbalances (Δq), resulting in membrane voltages of ∼ −50, −130, −380, and −610 mV. To study the effect of both voltage and ion gradients, we also generated a neutral ion concentration gradient of 9:1 between the extracellular and intracellular solutions, as similar gradients are often used in electrophysiology experiments (Fig. 1). All CompEL simulations were 500 ns long

TABLE 1 Summary of simulations performed in this study

Protein	Transmembrane voltage methodology	Voltage (mV)	Ion solution	Simulation duration (ns)
TRPM5	CompEL (antiparallel)	0	135:15 mM $NaCl/CaCl_2$	3×150
		−50	135:15 mM $NaCl/CaCl_2$	3×500
		−130	135:15 mM $NaCl/CaCl_2$	3×500
		−380	135:15 mM $NaCl/CaCl_2$	3×500
		−610	135:15 mM $NaCl/CaCl_2$	3×500
	external applied field	−50	150 mM NaCl	3×250
			150 mM $CaCl_2$	3×250
		−200	150 mM NaCl	3×250
			150 mM $CaCl_2$	3×250
		−340	150 mM NaCl	3×250
			150 mM KCl	3×250
			150 mM $CaCl_2$	3×250
TRPM5		−130	150 mM NaCl	3×250
F904T			150 mM $CaCl_2$	3×250
		−200	150 mM NaCl	3×250
			150 mM $CaCl_2$	3×250

and repeated three times for each system, resulting in an aggregated simulation time of 3 μs per individual channel and membrane voltage due to the double-channel nature of these simulations.

External applied field simulations

In addition to CompEL simulations, we also performed simulations in monocationic solutions of 150 mM NaCl, 150 mM KCl, and 150 mM $CaCl_2$, using an applied electric field to produce membrane voltage (48). Fields of −0.03, −0.0175, or −0.0044 V nm^{-1} were applied, resulting in transmembrane voltages of ∼–340, −200, or −50 mV, respectively, with negative polarity in the intracellular region. All applied field simulations were 250 ns long and repeated three times for each system.

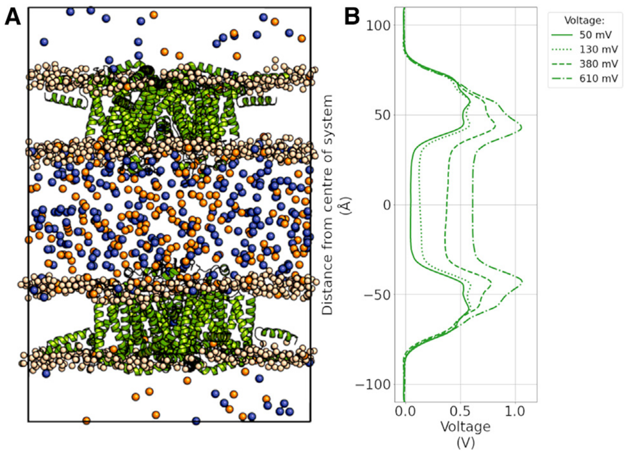

FIGURE 1 Structure and membrane voltage of CompEL simulations of TRPM5. (*A*) Snapshot of the CompEL system showing the TRPM5 pore domain of *Danio rerio* used in this study inserted into a double-bilayer simulation system in an antiparallel fashion so that both proteins experience identical voltage polarity. Cations within the aqueous compartments are shown as spheres (*orange*, calcium; *blue*, sodium), highlighting the 9:1 ion concentration gradient between the compartments. (*B*) The CompEL charge differences we applied across the aqueous compartments (Δq) resulted in transmembrane voltages of ∼ −50, −130, −380, and −610 mV in addition to the concentration gradient.

Umbrella sampling

Umbrella sampling was performed for each investigated ion type by placing a harmonic restraint on an individual ion in windows along the pore axis from the EPV to the intracellular channel exit with a spacing of 0.25 Å. The harmonic force constant used was 1000 kJ mol^{-1} nm^{-2}. Each window was simulated for 10 ns and the final potentials of mean force (PMFs) were determined by using the GROMACS utility gmx wham (49). The standard error of the mean was determined by block analysis of the simulation data.

Simulation analysis

Analysis of MD trajectory data was performed using in-house written Python scripts, utilizing GROMACS modules (30,31), the SciPy library of tools (50–53), and MDAnalysis (54,55). Analysis of the pore architecture was performed using CHAP (56). All plots were generated in Python using Matplotlib (57) and Seaborn (58). All MD input and analysis scripts used for this study are deposited in a public GitHub repository, available at: https://github.com/cmives/Na_selectivity_mechanism_of_TRPM_channels.

Calculating conductance and selectivity from in silico electrophysiology experiments

The conductance of the channels (C_{ion}) was calculated according to Eq. 1, where N_p is the number of permeation events, Q_{ion} is the charge of the permeating ion in Coulomb, t_{traj} is the length of the trajectory, and V_{tm} is the transmembrane voltage. The mean conductance and standard error were calculated from overlapping 50-ns windows of the trajectory.

$$C_{ion} = \frac{N_p \times Q_{ion}}{t_{traj} \times V_{tm}} \qquad (1)$$

TABLE 2 Calculated conductances and selectivities from CompEL simulations of ion permeation in the TRPM5 channel

		Conductance (pS)				
Conc. gradient	Voltage (mV)	Na^+	Ca^{2+}	Cl^-	Overall	P_{Na}/P_{Ca}
135:15 mM	0	0 ± 0 (15)	0 ± 0 (0)	0 ± 0 (0)	0 ± 0	n.d.
	−50	16 ± 3 (15)	0 ± 0 (0)	0 ± 0 (0)	16 ± 3	∞
	−130	7 ± 1 (18)	0 ± 0 (0)	0 ± 0 (0)	7 ± 1	∞
	−380	4 ± 1 (32)	5 ± 1 (19)	0 ± 0 (1)	9 ± 1	1.9 ± 0.4
	−610	10 ± 1 (115)	29 ± 3 (168)	−1 ± 0 (6)	38 ± 3	0.8 ± 0.1

Mean inward conductances and mean ± SE were calculated from overlapping 50 ns windows from threefold replicated 500 ns simulations of an antiparallel double-bilayer system. The number of permeation events associated with the conductance for each cation is displayed in parentheses. Mean selectivity ratios of Na^+ and Ca^{2+} permeation events and mean ± SE were calculated from threefold replicated 500 ns simulations (length of each 0 mV simulation: 150 ns; n.d., not determined).

The selectivity (P_{Na}/P_{Ca}) from the dicationic CompEL simulations was calculated as the ratio between the total sum of Na^+ permeation events and the total sum of Ca^{2+} permeation events across all simulations in a certain voltage or concentration regime.

Identification of cation binding sites from MD simulations

Cation binding sites were identified by plotting time series of each permeating ion with respect to their position along the pore axis. To further validate these positions, a 3D density mesh was generated for cations within 10 Å of the protein. This analysis was performed on a trajectory of concatenated, threefold replicated 500 ns simulations in monocationic solutions with a voltage of ∼ −50 mV produced by the CompEL method.

Characterizing permeation cooperativity through mutual information with SSI from PENSA

To characterize the level of cooperativity of the ion permeation mechanisms within the TRPM5 channel, we used PENSA to calculate the state-specific information (SSI) shared between discrete state transitions in the occupancy distributions of both of the pore binding sites (59,60). The methodology has been described in greater detail in our previous work (36).

In brief, a time series distribution with a time step of 20 ps for each binding site was obtained. For each frame, the ion's atom ID number was recorded if an ion occupied the binding site in this frame (occupied state). By contrast, if the binding site was unoccupied (vacant state), an ID of −1 was recorded. We then quantified by mutual-information whether ion transitions from occupied to vacant, or vice versa, at one site were coupled to similar ion transitions at the second ion binding site. To account for statistical noise that can arise from distributions even if they are uncorrelated with one another due to small-batch effects (61,62), we calculated a statistical noise threshold. This threshold level was subtracted from the measured SSI values to yield the excess mutual information, or excess SSI (exSSI) above noise.

RESULTS

Cation conductance of the TRPM5 channel in dicationic solutions

We performed in silico simulations of open state *Danio rerio* TRPM5 (24) embedded in a dual POPC lipid bilayer system, with a dicationic solution of 135 mM NaCl and 135 mM $CaCl_2$ in the central dense aqueous compartment, and 15 mM NaCl and 15 mM $CaCl_2$ in the outer diluted aqueous compartments, respectively (Fig. 1). An antiparallel CompEL double-bilayer setup (46) was used to yield a biomimetic transmembrane voltage of ∼ −50 mV across both embedded channels, as well as higher voltages of −130, −380, and −610 mV to increase the number of permeation events and improve the statistics of our analyses (Fig. 1). The 9:1 ion concentration gradient between the middle and the outside bulk compartment was expected to act synergistically with the membrane voltage to drive ion permeation.

Our simulations showed a continuous flow of permeating ions, resulting in a total of 374 permeation events across all investigated simulation conditions performed with the CompEL setup. The selectivity filter (SF) remained open throughout all of the simulations with a minimum filter diameter of 6 Å or greater (Fig. S1). Even though the ion gradient provided an additional driving force for permeation alongside the voltages, the calculated conductances from our in silico electrophysiology simulations were generally in good agreement with the published conductance values of 23–25 pS from in vitro electrophysiology experiments on TRPM5 in NaCl-based solutions (15,63). The experiments were conducted by recording current-voltage relationships of TRPM5 excised patches in solutions containing 140 mM NaCl and a small activating concentration of Ca^{2+} in voltage ranges from ∼ −100 to 100 mV. Within this range, at a membrane voltage of −50 mV, we observed a conductance of 16 ± 3 pS, derived from permeation counts using the CompEL protocol; in applied-field simulations (see below) we found a conductance of 19 ± 7 pS at this voltage (Table 2). Previous computational studies of Na^+ and K^+ permeation across ion channels have observed similar levels of agreement between experimental and simulated conductance values, with the CHARMM36m force field often showing the closest consensus (2,36,64). To ensure that the additional ion concentration gradient between the double bilayers did not influence the calculated conductance values, we carried out threefold replicated control simulations, in which the gradient was retained but no charge imbalance (voltage) was applied between the CompEL compartments. Within a total time of 450 ns, no ion permeated TRPM5 in these simulations (Table 2). It

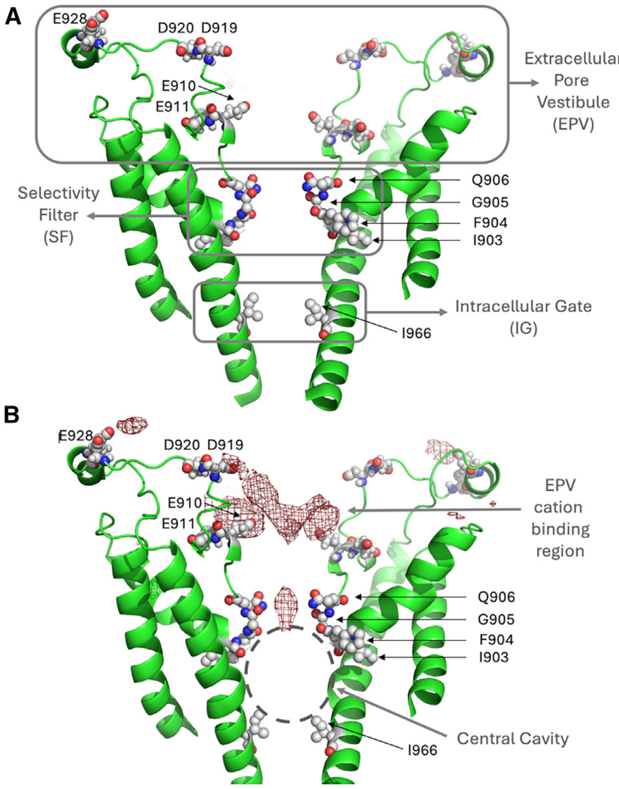

FIGURE 2 (*A*) Overview of the structure of the TRPM5 channel of *Danio rerio* used in this work (two of the four subunits are omitted for clarity). TRPM5 has a short, selectivity filter (SF) consisting mainly of Q906, G905, and F904. The hydrophobic intracellular gate (IG) of TRPM5 is formed by I966. In this study, the pore is defined as the region between the two constrictions of the channel, namely Q906 of the SF and I966 of the intracellular gate. Above the pore is the extracellular pore vestibule (EPV), which contains a number of acidic residues, such as E910, E911, D919, D920, and E928. Functionally important residues in the EPV, the SF, and the IG are shown as spheres and labeled. (*B*) 3D density map of Ca^{2+} ions in the TRPM5 channel. The density of Ca^{2+} ions was calculated from concatenated trajectories of TRPM5 in a dicationic solution under a transmembrane voltage of ~ -50 mV generated by the CompEL method. A major density maximum is seen within the EPV, where Ca^{2+} associates. Occasionally, Ca^{2+} ions migrated into the SF (minor density maximum at Q906); however, they were not able to traverse past the SF at biomimetic voltages. Functionally important residues in the EPV, the SF, and the IG are shown as spheres and labeled.

can therefore be concluded that ion permeation is predominantly voltage driven on the timescale of the simulations.

Low-voltage simulations in dicationic solutions show exclusive permeation of Na^+ through TRPM5

At the lowest simulated voltages of ~ -50 and -130 mV, we observed complete Na^+ selectivity in mixed Ca^{2+}/Na^+ solutions, with no recorded Ca^{2+} permeation during an accumulated simulation time of 1.5 μs. During the same timespan, 15 (-50 mV) and 18 Na^+ ions (-130 mV) traversed the TRPM5 pore, respectively, in accordance with its general conductance level (Table 2).

Analysis of the pore architecture of TRPM5 showed no major conformational changes during the course of the simulations (for root mean-square deviation and fluctuation during the simulations, see Fig. S2). The TRPM5 pore possesses two main constrictions: an upper constriction formed by the sidechains of Q906 and by G905 of the short SF, and a lower constriction formed by the sidechains of I966 of the intracellular gate (Figs. 2 *A* and 3 *A*). A minor constriction can also be observed $\sim$ 13 Å above the SF, in the extracellular pore vestibule (EPV) (Figs. 2 *A* and 3 *A*). This constriction is formed by the turret loop between the pore helix and the S6 helix. The water sites near Q906 were occupied by water molecules during most of the simulated time (Fig. S3).

In our simulations at -50 and -130 mV, Na^+ cations first entered the EPV region of the TRPM5 pore, where they showed a broad association with the protein matrix. Permeating Na^+ cations then traversed the SF rapidly, and entered the pore cavity. They spent a substantial amount of time occupying the cavity before passing through the lower gate and exiting the pore at the intracellular face.

As opposed to monovalent Na^+, Ca^{2+} ions did not readily enter the inner pore of TRPM5 during the course of the simulations. Ca^{2+} cations chiefly occupied the EPV region at the extracellular entrance (see Fig. 2 *A*). 3D density maps of Na^+ and Ca^{2+} ions further confirmed this observation (Fig. 2 *B*). The maps show substantial Ca^{2+} density in the EPV, particularly near the acidic residues on the loop between the pore helix and the S6 helix, namely: E910, E911, D919, D920, and E928. We observed that Ca^{2+} ions occasionally migrated from the EPV toward the pore; however, they were blocked from entering the cavity at the SF, particularly at the constriction formed around residues G905 and Q906 from each subunit (Fig. 2 *B*).

Voltage dependence of simulated TRPM5 ion selectivity

As the membrane voltage was increased in the CompEL simulations, we observed the Na^+ selectivity (P_{Na}/P_{Ca}) to be diminished (Table 2). At a voltage of both ~ -50 and ~ -130 mV, we recorded complete Na^+ selectivity, with no Ca^{2+} permeation events in any of the simulations. At a voltage of ~ -380 mV, the in silico electrophysiology simulations continued to display slightly Na^+-selective permeation; however, when the voltage was further increased to ~ -610 mV, the Na^+ selectivity was abolished (see also Fig. S4). Furthermore, higher-voltage simulations also yielded a small number of Cl^- permeation events, with anions permeating through to the extracellular solution.

Our findings suggest relatively weak cation binding sites within the pore domain, in line with the absence of negatively charged residues lining the SF and inner cavity,

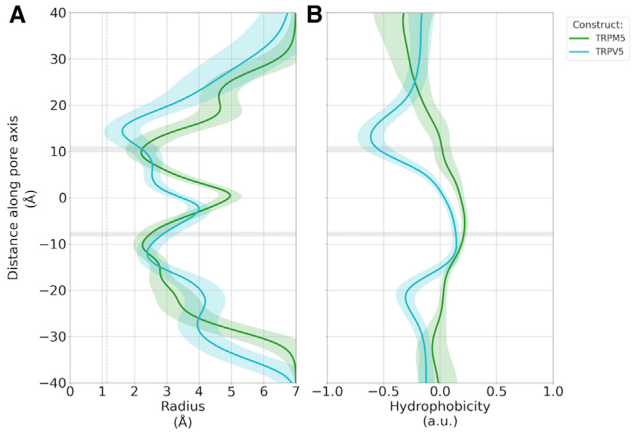

FIGURE 3 Pore architecture of the monovalent-selective TRPM5 channel (*green*) and the Ca^{2+}-selective TRPV5 channel (*cyan*) from MD simulations. The average pore radius (*A*) and hydrophobic profile (*B*) for each channel was calculated using CHAP (56). The standard deviation is shown as shaded regions. The profile of the TRPV5 channel was generated from simulation data published previously (36). The shaded gray regions represent the average position of the pore constrictions formed by Q906 in the SF (*upper*) and I966 of the intracellular gate (*lower*) in TRPM5. The z = 0 position in this and the following figures refers to the center of the membrane in z-dimension; negative positions on the axis point into the direction of the intracellular side, while positive positions point in the direction of the extracellular side of the membrane relative to the center.

because of the substantial effect of supraphysiological membrane voltages on selectivity. To further explore the ion permeation mechanism in TRPM5 and their underlying determinants, we thus aimed to enhance the sampling of both Na^+ and Ca^{2+} permeation, while at the same time remain within the Na^+-selective voltage regime. We selected an intermediate voltage of ~ -340 mV for investigating permeation in monocationic solutions to ensure a sufficient number of traversals of both Ca^{2+} and Na^+ ions in the monovalent-selective regime.

Mechanistic insights into ion permeation in TRPM5 from monocationic solutions

We conducted in silico electrophysiology simulations with an applied electric field, generating a membrane voltage of ~ -340 mV, to investigate the permeation mechanism of Na^+, K^+, and Ca^{2+} ions in monocationic solutions at sufficient sampling efficiency (Table 3). As shown in Fig. 4, we observed a clear difference between the behavior of the monovalent Na^+ and K^+ ions in the channel and the divalent Ca^{2+} ions, especially near and in the central cavity.

As can be seen, whereas Na^+ and K^+ ions occupied the central cavity of the channel for most of the simulated time, permeating Ca^{2+} ions traversed the inner cavity rapidly, not exhibiting any obvious immobilization within the cavity. Despite occupying the cavity for extended periods of time, Na^+ and K^+ ions did not seem to bind to a particular binding position or residue within the cavity, but

TABLE 3 Calculated conductances from applied field simulations of ion permeation in the TRPM5 channel

Voltage (mV)	Ion solution	Conductance (pS)
−50	150 mM NaCl	19 ± 7 (4)
	150 mM $CaCl_2$	0 ± 0 (0)
−200	150 mM NaCl	17 ± 3 (15)
	150 mM $CaCl_2$	9 ± 4 (4)
−340	150 mM NaCl	52 ± 6 (83)
	150 mM KCl	21 ± 4 (34)
	150 mM $CaCl_2$	85 ± 5 (54)

Mean inward conductances and mean ± SE were calculated from overlapping 50 ns windows from threefold replicated 500 ns simulations of a single bilayer system. The number of permeation events observed for each cation is displayed in parentheses.

instead explored nearly the entire cavity volume before they permeated through the lower gate to the intracellular side. In this way, the cavity served to store a monovalent ion rather than providing specific binding sites for it. A similar behavior has recently been described for simulations of Na^+ ions in the cavity of the homodimeric, endolysosomal Na^+-selective cation channel TPC2 (65).

Looking at the density of ions along the pore axis, and using the negative logarithmic density as an estimate for the underlying multiion free energy profile at the examined nonequilibrium permeation conditions under a membrane voltage of −340 mV, it can be observed that the cavity region formed only a shallow, broad energy minimum for the permeating monovalent cations, whereas, in contrast, permeating Ca^{2+} ions experienced a small apparent energy barrier in the same region (Fig. 5 *A*). Both monovalent and divalent ions showed further binding to a relatively shallow binding site at the EPV. In addition, all ion types experienced a slight energy barrier to translocation near the intracellular channel exit (hydrophobic lower gate). Notably, the ions did not show major interactions with the SF in this analysis. This observation, again, is in accordance with observations made in simulations of TPC2 (65).

In addition to the nonequilibrium free energy estimates, we calculated single-ion PMFs under equilibrium conditions for each ion type by umbrella sampling. Moving into the SF from the EPV, all ions experience a small energy barrier in the SF before Na^+ and Ca^{2+} interact favorably with Q906 and G905, whereas K^+ shows limited interactions there. When traversing further into the cavity, both monovalent ion types experience a broad relative energy minimum across the entire length of the cavity. By contrast, the divalent Ca^{2+} ions encounter a substantial energy barrier upon entering the cavity near the hydrophobic residues F904 and I903. Notably, the whole cavity forms an unfavorable region for Ca^{2+}. Migrating through the intracellular hydrophobic gate leads to a considerable energy barrier for both of the monovalent ion types, which is raised even further for Ca^{2+} ions. The higher gate barrier seen here as compared with the logarithmic density energy estimate is likely due

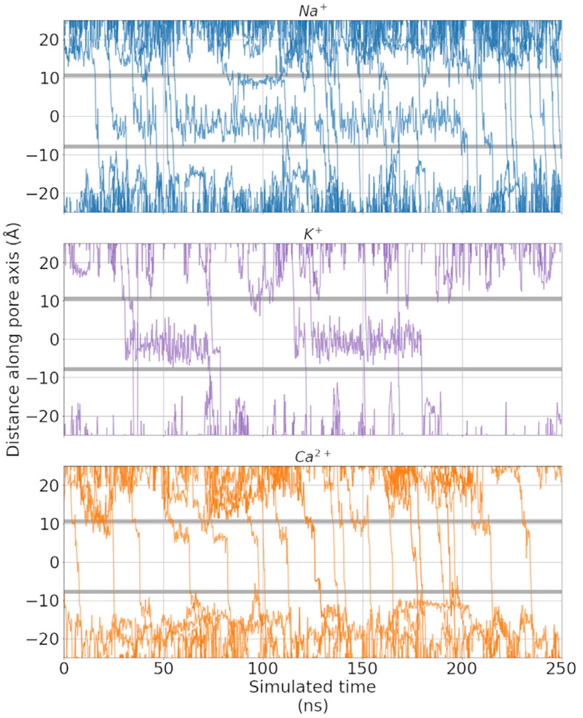

FIGURE 4 Exemplar permeation traces of the *z*-coordinate of permeating Na^+ (*blue*, *top*), K^+ (*purple*, *middle*), and Ca^{2+} (*orange*, *bottom*) over time, plotted from simulations performed in a monocationic solution with an applied electric field (−340 mV). The shaded gray regions represent the average position of the pore constrictions formed by Q906/G905 in the SF (*upper*) and I966 of the hydrophobic gate (*lower*). Please note, only cations that fully permeate through the pore within the 250 ns simulations are shown in the plot. Exemplar videos of permeating ions are available at: https://github.com/cmives/Na_selectivity_mechanism_of_TRPM_channels.

to the absence of multi-ion effects in the single-ion PMFs (see below).

We also conducted additional simulations using applied external electric fields of differing magnitudes. Similar to the low-voltage CompEL simulations, whereas the main features of the ion density and free energy estimates occurred across all tested voltages, Ca^{2+} was increasingly excluded from the cavity at these lower voltages, and no longer able to enter into the cavity at the lowest voltage of −50 mV during the timespan of our simulations (Fig. S4). This again showed that the ion selectivity of TRPM5 was voltage dependent in the simulations. We therefore next aimed to elucidate the molecular foundations of this behavior and, importantly, the ion selectivity of TRPM5 in general.

Solvation profiles of cations during channel permeation

To probe if cation desolvation played a part in selective ion permeation, we calculated the number of water oxygen atoms within the first solvation shell around the ions (Fig. 5). Among other mechanisms (36,67), the desolvation of permeating ions has previously been reported to represent an important potential selectivity mechanism in ion channels (2,68). Differences in the desolvation energies of permeating ions provide a thermodynamic penalty which can underpin the more favorable permeation of an ionic species over another. Here, the free energy required to desolvate Ca^{2+} strongly exceeds that for Na^+ and K^+ (69), such that this difference could give rise to monovalent selectivity in TRPM5.

In the bulk solution of the simulated systems, Na^+, K^+, and Ca^{2+} ions showed the expected water coordination number of their solvation shells. As both Na^+ and K^+ ions entered the pore of TRPM5, they became partially desolvated within the SF. The SF matrix displaced, on average, one water molecule from the first solvation shell of Na^+ and two water molecules from the first solvation shell of K^+ ions. Full resolvation of Na^+ and K^+ occurred about 10 Å farther toward the intracellular side, in the center of the cavity, but not immediately after exiting the SF. At the intracellular hydrophobic gate formed near I966, a level of desolvation similar to the SF was observed for both Na^+ and K^+. By contrast, the rapidly permeating Ca^{2+} ions did not show any level of desolvation when they crossed the SF, cavity, or intracellular hydrophobic gate of TRPM5.

These findings suggest that ion desolvation in the SF or inner pore is not a major factor in achieving selectivity for monovalent cations. Ca^{2+} ions were not desolvated when they traversed the inner cavity. As the energetic penalty for desolvating Ca^{2+} is much larger than for Na^+ or K^+ (69), the observed desolvation profiles therefore suggest that desolvation does not underpin the deselection of Ca^{2+} ions in TRPM5.

Why does the central cavity form an attractive site for monovalent cations but a repulsive site for divalent cations?

The presence of a water-filled internal cavity is a conserved feature among cation-selective channels. The cavity serves to maintain a high degree of ion hydration despite locating to the center of the hydrophobic lipid bilayer, and to focus the membrane voltage difference onto the SF (70). Like in other monovalent-selective cation channels such as TPC2 (65), we observed in TRPM5 that the major permeating species, Na^+ and K^+ ions, were rehydrated and transiently captured in the cavity after their permeation through the SF (Figs. 4 and 5). Despite their extended residence time in the cavity, we did not observe the ions to bind to specific binding sites within the cavity. This observation is reflected in the single-ion PMF profiles, where the cavity displays a mostly flat free energy profile without significant binding sites for the monovalent ions (Fig. 6). Ca^{2+} ions were able to enter and traverse the cavity at higher voltages, but did not alter their hydration number during this process. This suggests, again, that they did not interact favorably with

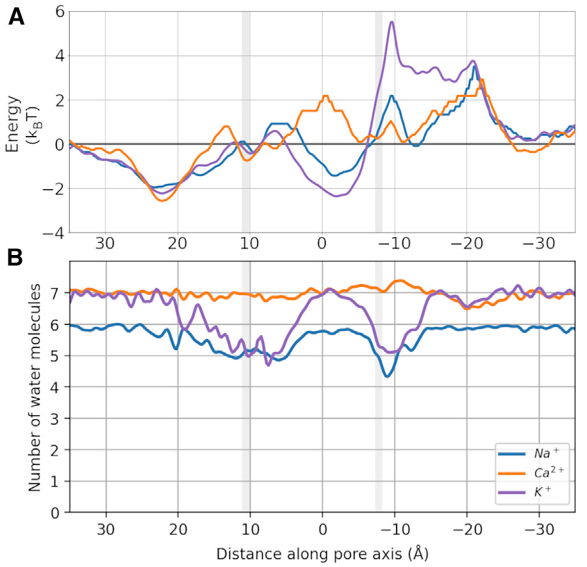

FIGURE 5 Log-density and solvation profiles of Na^+ (*blue*), K^+ (*purple*), and Ca^{2+} (*orange*) cations in the TRPM5 pore from simulations with a monocationic solution, and an applied transmembrane voltage of ~ -340 mV. (*A*) Negative logarithmic density profiles of permeating cations as estimates of the nonequilibrium energy surface the ions experience in the pore (energy unit: k_BT). Minima reflect binding sites, while maxima indicate barriers between the binding sites. The location of the pore constrictions formed by Q906 (*upper*) and I966 (*lower*) are shown as gray regions. (*B*) The mean number of oxygen atoms of water molecules in the first solvation shell of each permeating cation is shown. Radii used were: Na^+, 3.1 Å (66); K^+, 3.5 Å; and Ca^{2+}, 3.0 Å (37). The curves in both plots have been smoothed using a Gaussian filter with a sigma value of 2.

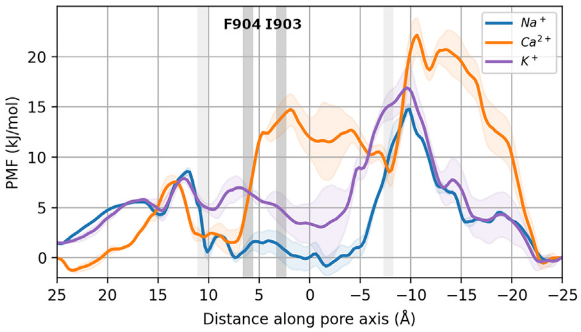

FIGURE 6 Potentials of mean force (PMFs) for the three ion types traversing the TRPM5 pore. The location of the pore constrictions formed by Q906 (*upper*) and I966 (*lower*) are shown in light gray, the location of the hydrophobic residues F904 and I903 in the transition zone between the SF and cavity in dark gray. The error ranges show standard errors of the mean.

any of the cavity-lining residues or the cavity overall. At lower voltages, by contrast, Ca^{2+} ions were completely excluded from entering the cavity. Accordingly, the single-ion PMF profile for Ca^{2+} shows no favorable interactions between Ca^{2+} and the cavity for all of its extent.

We therefore investigated the difference between the pore and cavity properties of the highly Ca^{2+}-selective TRP channel, TRPV5, and the monovalent-selective TRPM5. Contrary to TRPM5, the cavity of TRPV5 shows a high occupancy with Ca^{2+} ions (36). As displayed in Fig. 3, the general features of the pore are preserved with a constriction at the extracellular SF, a wider internal cavity region, and a second constriction at the intracellular gate. TRPM5 has a markedly shorter SF, while its cavity is wider than that of TRPV5. However, there is a substantial difference in the pore lining of the two TRP channels. Whereas the TRPV5 SF is a strongly hydrophilic region, TRPM5 does not exhibit increased hydrophilicity within its SF. The transition from the SF to the cavity is slightly hydrophobic in TRPM5, while this is a hydrophilic region in TRPV5. There are no differences between the hydrophobicity of the two channels at the intracellular gates. The hydration level of the channel shows a decrease in the SF and transition zone along with the intracellular gate (Fig. S5). No dewetting or vapor phase transitions indicative of hydrophobic gating were observed, however (71–73).

The differing properties of the inner pore (cavity and SF) suggest that the absence of favorable interactions of TRPM5 with Ca^{2+} in this region arises due to the raised hydrophobicity of its SF and upper portion of its inner cavity (hydrophobic funnel). In particular, the transition zone between the SF and the cavity in TRPM5 is lined by large hydrophobic residues at the bottom of the SF, especially F904 and I903. This sequence is shared with TRPM4, which is also a monovalent cation-selective channel (Fig. 7 *A*). On the pore-forming helix S6 of TRPM5, the additional hydrophobic residues V959 and L962 line the cavity toward the hydrophobic lower gate at I966, whereas only two polar side chains, N958 and N962, are involved. The conservation level of the large hydrophobic residues lining the cavity is generally high (*dark* and *light pink surface color* in Fig. 7 *B*).

The energetic cost of placing monovalent cations into a hydrophobic environment is smaller than that for divalent cations, even when they retain their hydration shells. The Born energy for divalent cations, for example, quantifying this electrostatic energy penalty in continuum models, is four times larger than the penalty associated with monovalent cations (78). As observed in the PMFs and the simulations under increased voltage, the protein matrix does not form favorable interactions with Ca^{2+} ions in its inner pore. The hydrophobicity of the TRPM5 pore gradually increases along the pore axis, with only few hydrophilic sites within the SF or central cavity.

Our results thus suggest that Ca^{2+} ions are unable to enter the increasingly hydrophobic environment of this region at physiological voltages, and only penetrate past the SF under high-voltage conditions, above the physiologically relevant level.

Selectivity for monovalent cations is linked to permeation cooperativity between two binding regions

We hypothesized that, due to the presence of an additional binding or "storage" region for monovalent cations in the

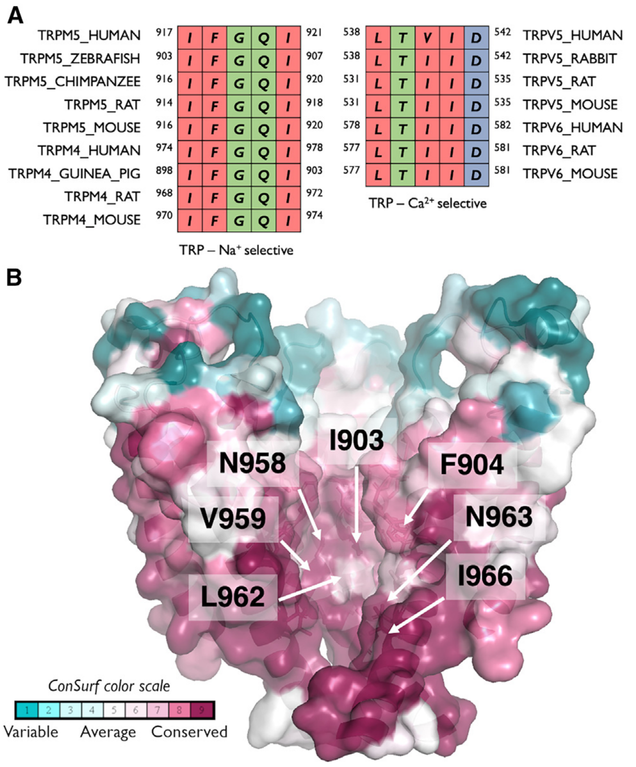

FIGURE 7 (*A*) Sequence conservation of the SF and transition zone to the inner cavity in the monovalent-selective channels TRPM5 and TRPM4 compared with the Ca^{2+}-selective channels TRPV5 and TRPV6. Colors according to Clustal Omega convention (74). (*B*) Evolutionary conservation of the pore cavity in TRPM5 channels. Evolutionary conservation scores were calculated using ConSurf (75). A multiple sequence alignment of 520 sequences was built of TRPM4 and TRPM5 sequences from UniProt (76). The evolutionary conservation scores were projected onto the structure of TRPM5 from *Danio rerio*, with one subunit removed for clarity. Figure made with PyMOL (77).

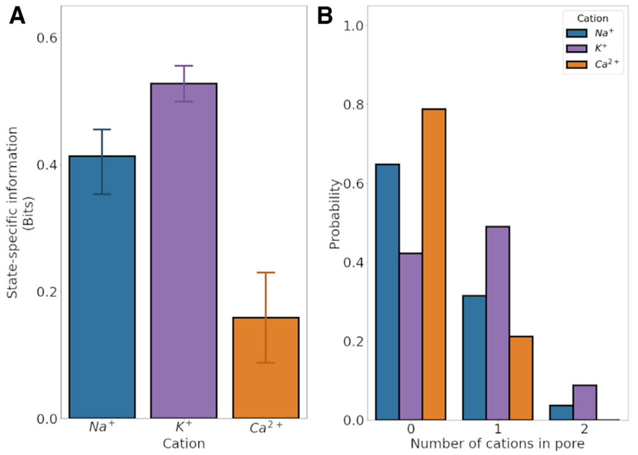

FIGURE 8 SSI of cation transitions between binding sites and the average number of each cation within the pore of TRPM5. (*A*) exSSI between the EPV and pore cavity ion binding sites, quantifying the degree of cooperativity in the permeation mechanisms of Na^+ (*blue*), K^+ (*purple*), and Ca^{2+} (*orange*) ions. The mean exSSI and mean $\pm$ SE between transitions from the two binding sites were calculated from simulations performed in monocationic solutions with an externally applied voltage of ~ -340 mV. (*B*) Mean probability for the number of each cationic species within the inner TRPM5 pore, calculated from nonoverlapping 50 ns windows from threefold replicated 250 ns simulations. We defined the inner pore as the region between the constrictions formed by Q906 in the SF and I966 at the lower hydrophobic gate.

internal cavity compared with Ca^{2+}, the permeation mechanism for monovalent ions may be more efficient than for Ca^{2+}. In previous work, we developed a mutual-information-based quantification method of the level of cooperativity when ions permeate across multiple ion channel binding sites, termed SSI (36,58,59). In brief, SSI quantifies the probability that a state change of one binding site, such as a change from binding an ion to becoming vacant upon ion permeation, is correlated to a similar state change in a second binding site, in which case the unbinding events are coupled to one another, for instance by a knock-on mechanism.

We applied the SSI approach to ion conduction in TRPM5, focusing on the pair of ion binding regions at the EPV and the channel cavity (see Fig. 2). These two binding areas are shallow and relatively distant to one another, but locate directly to the main pore axis. In addition, they show moderate to high occupancy with monovalent cations, respectively (Fig. 8).

By using SSI, we found that both Na^+ and K^+ ions displayed a high level of correlation between binding and unbinding at the two successive sites, whereas the permeation of Ca^{2+} ions showed only a low degree of correlation slightly above the noise level (Fig. 8). This suggests that a distant knock-on mechanism is in operation between incoming monovalent ions, which, when unbinding from the EPV, bind transiently at the SF, as well as over substantial timespans within the cavity. In other words, the cavity serves as a reservoir, more likely to release a Na^+ or K^+ ion to the cytoplasm when a further monovalent cation approaches and inserts into the TRPM5 SF.

By contrast, Ca^{2+} ions permeated on their own. Their traversal is likely to be driven solely by the supraphysiological transmembrane electric field. Accordingly, lower-voltage simulations did not show any permeating Ca^{2+}. This selectivity for monovalent ions was abolished when the driving force for Ca^{2+} permeation exceeded a certain threshold. Permeation at higher voltages could thus be described as a "pull-through" of Ca^{2+} ions across the otherwise unfavorable environment of the inner cavity for divalent ions. This voltage-driven pull-through occurs due to the higher charge of Ca^{2+}, doubling the Coulombic driving force compared with the monovalent ions, but is unlikely to be physiological.

Our results suggest that the occurrence of two binding regions for monovalent ions, at the EPV and in the central cavity forming a reservoir site, enhances permeation efficiency via a distant knock-on mechanism. By contrast, Ca^{2+} ions are prevented from entering the pore cavity at physiologically relevant membrane voltages by a hydrophobic gate

region, abolishing the reservoir binding within and thus disrupting any sizable permeation cooperativity.

The transition zone from the SF to the central cavity is key for monovalent cation selectivity in TRPM5

To test the hypothesis that the hydrophobicity of the transition zone connecting the SF to the central cavity provides the primary barrier to the permeation of divalent cations, we performed applied-field simulations of TRPM5 with a F904T mutation, which increases the hydrophilicity of the main barrier area, at two voltage levels. In the Ca^{2+}-selective TRPV channels TRPV5 and TRPV6, a threonine residue is located at the structurally equivalent position. Since they also contain a charged glutamate in their SF, which shows a high affinity for Ca^{2+} but is absent in TRPM5, we did not expect the TRPM5 F904T mutant to show a similarly high degree of Ca^{2+} selectivity. Rather, we hypothesized that the added hydrophilicity of the mutant may reduce the height of the hydrophobic barrier for both monovalent and divalent cations, that is, increase the flow of Na^+ while allowing the passage of Ca^{2+} in physiologically relevant voltage ranges. It is known that the effect of single-point mutations is not always restricted to the specific residue mutated. In this case, mutation from F to T could be expected to change the pore size, may add an additional ion interaction site, and change the packing of the protein. However, since the F side chain does not constrict the pore further and largely points away from the main ion conduction pathway, we expect that the major effect of the F904T mutation is due to a change in hydrophobicity in this area.

Threefold replicated simulations of the F904T mutant in 150 mM $CaCl_2$ at -130 mV showed 13 completed Ca^{2+} permeation events within a total time of 750 ns. In the same timespan, 27 Na^+ ions traversed the mutant channel in 150 mM NaCl solution (Table S3). The permeation numbers correspond to a selectivity of P_{Na}/P_{Ca} of ~ 2, otherwise not observed below a voltage of -380 mV. Additional control simulations at -200 mV in monocationic solution displayed 15 Na^+ and 4 Ca^{2+} permeation events in the WT (Table S2), whereas the mutant conducted 88 Na^+ ions and 28 Ca^{2+} ions within the same accumulated timespan (Table S3).

These results show that the F904T mutation indeed facilitated the permeation of Ca^{2+}, while at the same time increasing the Na^+ flux. We therefore conclude that the hydrophobic transition zone between the SF and the central cavity plays the major role in governing the selectivity of TRPM5 for monovalent cations at physiological voltages.

DISCUSSION

The TRP channel superfamily encompasses a broad range of cation-selective ion channels of great physiological and biomedical importance (5,6). While most members of the superfamily translocate both monovalent cations and divalent Ca^{2+} at similar permeabilities, TRPV5 and TRPV6 are strongly Ca^{2+} selective (36,37,79) and TRPM4 and TRPM5 are selective for monovalent cations (15,16). In contrast to most other Na^+-selective channels (1), the SFs of TRPM4, TRPM5, and the endolysosomal TPC2 do not contain charged residues, while they show a relatively high abundance of hydrophobic residues (24,65).

Ion selectivity is usually linked to the presence of specific binding sites in the channels' SF and inner pore (80,81), cooperativity between the permeation kinetics of multiple such binding sites (36,80,82,83), ion desolvation (2,68), or size exclusion-effects (1,80). Our simulations showed, however, that no high-affinity binding sites for cations exist within the pore of TRPM5, that only minor ion desolvation effects occur, and that size exclusion does not play a key role in selectivity, as the channel can conduct both monovalent and divalent cations at slightly increased membrane voltages. We observed two shallow, broad ion binding regions for monovalent cations in the TRPM5 channel; one within the EPV above the SF, and a second within the central cavity, whereas divalent cations did not interact favorably within the cavity.

Our findings suggest a new mechanism of monovalent cation selectivity, in which the combination of an uncharged, relatively hydrophobic SF and the presence of large hydrophobic side chains at the entrance to the central channel cavity determine the monovalent selectivity of TRPM5. Since the traversal of a divalent cation through this hydrophobic funnel incurs a larger energy penalty as compared with a monovalent cation, this hydrophobic region creates a higher energy barrier for the permeation of divalent cations (78). The hydrophobic energy barrier difference generated in this way is of moderate magnitude, and therefore can be overcome by divalent cations in the supraphysiological voltage range.

Under physiologically relevant voltages, this barrier and the generally largely hydrophobic character of the central cavity prevent divalent cations from entering and residing within the central cavity, whereas monovalent cations readily enter and occupy the cavity over substantial timespans. Numerous closed-state structures of TRPM4, a close monovalent-selective homolog of TRPM5, have been published within the PDB. Several of these structures include Na^+ cations modeled within the pore cavity (84,85). Consequently, these structures (PDB: 6BCJ, 6BCL, and 6BWI) suggest that the presence of a monovalent cation binding region in the inner cavity is a conserved feature among the monovalent-selective TRPM4 and TRPM5 channels.

Due to this additional binding region, a distant knock-on mechanism is established between an incoming monovalent cation and the monovalent cation stored within the cavity, which greatly increases permeation efficiency. By contrast, under supraphysiological voltage conditions, divalent cations are simply pulled through the hydrophobic cavity on

their own, exhibiting no interactions with the cavity matrix or other cations. Accordingly, our application of the mutual-information-based SSI method (36,86) to ions permeating through TRPM5 showed only negligible cooperativity for Ca^{2+} permeation.

Finally, we examined whether a hydrophilic mutation at the entrance to the central cavity facilitated the flux of divalent cations through this region. According to the findings discussed above, this was indeed the case in the F904T mutant of TRPM5, with substantial Ca^{2+} permeation in a voltage range that did not allow for Ca^{2+} permeation in the WT channel. In line with a hydrophobic barrier that exists for both monovalent and divalent cations, but is larger for divalent cations, the flow of Na^{+} also increased in the mutant, raising its overall conductance level.

DATA AND CODE AVAILABILITY

MD simulation inputs and analysis scripts used for this study are deposited in a public GitHub repository, available at: https://github.com/cmives/Na_selectivity_mechanism_of_TRPM_channels.

SUPPORTING MATERIAL

Supporting material can be found online at https://doi.org/10.1016/j.bpj.2024.07.035.

ACKNOWLEDGMENTS

We thank the University of Dundee I.T. services for maintenance of the School of Life Sciences high-performance computing (HPC) cluster which was utilized in this research. C.M.I. was supported by the UKRI - Medical Research Council (grant no. MR/N013735/1), N.J.T. and A.T.Ş. were supported by the UKRI - Biotechnology and Biological Sciences Research Council (BBSRC) (grant nos. BB/M010996/1 and BB/T00875X/1, respectively).

AUTHOR CONTRIBUTIONS

U.Z. and C.M.I. conceived the idea and designed the computational study. C.M.I. conducted and C.M.I. and U.Z. analyzed the WT simulation data. A.T.Ş. conducted and analyzed the F904T mutant simulations. N.J.T. analyzed the SSI data. U.Z. supervised the work. C.M.I. and U.Z. wrote the manuscript with contributions from N.J.T., and all authors edited and reviewed the manuscript.

DECLARATION OF INTERESTS

The authors declare no competing interests.

REFERENCES

1. Dudev, T., and C. Lim. 2014. Ion selectivity strategies of sodium channel selectivity filters. *Acc. Chem. Res.* 47:3580–3587.
2. Kopec, W., D. A. Köpfer, …, U. Zachariae. 2018. Direct knock-on of desolvated ions governs strict ion selectivity in K^{+} channels. *Nat. Chem.* 10:813–820. https://doi.org/10.1038/s41557-018-0105-9.
3. Roux, B. 2017. Ion channels and ion selectivity. *Essays Biochem.* 61:201–209.
4. Caterina, M. J., M. A. Schumacher, …, D. Julius. 1997. The capsaicin receptor: a heat-activated ion channel in the pain pathway. *Nature.* 389:816–824. https://doi.org/10.1038/39807.
5. Khalil, M., K. Alliger, …, M. A. Engel. 2018. Functional role of transient receptor potential channels in immune cells and epithelia. *Front. Immunol.* 9:174.
6. Ramsey, I. S., M. Delling, and D. E. Clapham. 2006. An introduction to TRP channels. *Annu. Rev. Physiol.* 68:619–647. https://doi.org/10.1146/annurev.physiol.68.040204.100431.
7. Nilius, B. 2007. TRP channels in disease. *Biochim. Biophys. Acta.* 1772:805–812.
8. Koivisto, A. P., M. G. Belvisi, …, A. Szallasi. 2022. Advances in TRP channel drug discovery: From target validation to clinical studies. *Nat. Rev. Drug Discov.* 21:41–59.
9. Moran, M. M. 2018. TRP Channels as Potential Drug Targets. *Annu. Rev. Pharmacol. Toxicol.* 58:309–330. https://doi.org/10.1146/annurev-pharmtox-010617-052832.
10. Nilius, B., and G. Owsianik. 2011. The transient receptor potential family of ion channels. *Genome Biol.* 12:218.
11. Samanta, A., T. E. T. Hughes, and V. Y. Moiseenkova-Bell. 2018. Transient receptor potential (TRP) channels. *Subcell. Biochem.* 87:141–165.
12. van Goor, M. K., L. de Jager, …, J. van der Wijst. 2020. High-resolution structures of transient receptor potential vanilloid channels: Unveiling a functionally diverse group of ion channels. *Protein Sci.* 29:1569–1580.
13. Hilton, J. K., M. Kim, and W. D. Van Horn. 2019. Structural and Evolutionary Insights Point to Allosteric Regulation of TRP Ion Channels. *Acc. Chem. Res.* 52:1643–1652. https://doi.org/10.1021/acs.accounts.9b00075.
14. Goretzki, B., C. Guhl, …, U. A. Hellmich. 2021. Unstructural biology of TRP ion channels: the role of intrinsically disordered regions in channel function and regulation. *J. Mol. Biol.* 433:166931.
15. Hofmann, T., V. Chubanov, …, C. Montell. 2003. TRPM5 is a voltage-modulated and Ca2+-activated monovalent selective cation channel. *Curr. Biol.* 13:1153–1158.
16. Owsianik, G., K. Talavera, …, B. Nilius. 2006. Permeation And Selectivity Of TRP Channels. *Annu. Rev. Physiol.* 68:685–717. https://doi.org/10.1146/annurev.physiol.68.040204.101406.
17. Ullrich, N. D., T. Voets, …, B. Nilius. 2005. Comparison of functional properties of the Ca2+-activated cation channels TRPM4 and TRPM5 from mice. *Cell Calcium.* 37:267–278.
18. Liu, D., and E. R. Liman. 2003. Intracellular Ca2+ and the phospholipid PIP2 regulate the taste transduction ion channel TRPM5. *Proc. Natl. Acad. Sci. USA.* 100:15160–15165.
19. Pérez, C. A., L. Huang, …, R. F. Margolskee. 2002. A transient receptor potential channel expressed in taste receptor cells. *Nat. Neurosci.* 5:1169–1176.
20. Zhang, Y., M. A. Hoon, …, N. J. P. Ryba. 2003. Coding of sweet, bitter, and umami tastes: different receptor cells sharing similar signaling pathways. *Cell.* 112:293–301.
21. Brixel, L. R., M. K. Monteilh-Zoller, …, D. Prawitt. 2010. TRPM5 regulates glucose-stimulated insulin secretion. *Pflügers Archiv.* 460:69–76.
22. Colsoul, B., A. Schraenen, …, R. Vennekens. 2010. Loss of high-frequency glucose-induced Ca2+ oscillations in pancreatic islets correlates with impaired glucose tolerance in Trpm5-/- mice. *Proc. Natl. Acad. Sci. USA.* 107:5208–5213.
23. Vennekens, R., M. Mesuere, and K. Philippaert. 2018. TRPM5 in the battle against diabetes and obesity. *Acta Physiol.* 222:e12949.

24. Ruan, Z., E. Haley, …, W. Lü. 2021. Structures of the TRPM5 channel elucidate mechanisms of activation and inhibition. *Nat. Struct. Mol. Biol.* 28:604–613. https://doi.org/10.1038/s41594-021-00607-4.

25. Jo, S., T. Kim, …, W. Im. 2008. CHARMM-GUI: A web-based graphical user interface for CHARMM. *J. Comput. Chem.* 29:1859–1865. https://doi.org/10.1002/jcc.20945.

26. Jo, S., X. Cheng, …, W. Im. 2014. CHARMM-GUI PDB manipulator for advanced modeling and simulations of proteins containing nonstandard residues. *Adv. Protein Chem. Struct. Biol.* 96:235–265. https://doi.org/10.1016/bs.apcsb.2014.06.002.

27. Lomize, M. A., I. D. Pogozheva, …, A. L. Lomize. 2012. OPM database and PPM web server: Resources for positioning of proteins in membranes. *Nucleic Acids Res.* 40:370–376. https://doi.org/10.1093/nar/gkr703.

28. Jo, S., T. Kim, and W. Im. 2007. Automated builder and database of protein/membrane complexes for molecular dynamics simulations. *PLoS One.* 2:e880. https://doi.org/10.1371/journal.pone.0000880.

29. Wu, E. L., X. Cheng, …, W. Im. 2014. CHARMM-GUI membrane builder toward realistic biological membrane simulations. *J. Comput. Chem.* 35:1997–2004. https://doi.org/10.1002/jcc.23702.

30. Abraham, M. J., T. Murtola, …, E. Lindahl. 2015. GROMACS: High performance molecular simulations through multi-level parallelism from laptops to supercomputers. *SoftwareX.* 1–2:19–25. https://doi.org/10.1016/j.softx.2015.06.001.

31. Lindahl, A., and S. van der Hess. 2020. GROMACS 2020.2 Source Code. https://doi.org/10.5281/zenodo.3773801.

32. Zhang, A., H. Yu, …, C. Song. 2020. The Ca^{2+} permeation mechanism of the ryanodine receptor revealed by a multi-site ion model. *Nat. Commun.* 11:922. https://doi.org/10.1038/s41467-020-14573-w.

33. Liu, C., A. Zhang, …, C. Song. 2021. Atomistic details of charge/space competition in the Ca2+selectivity of ryanodine receptors. *J. Phys. Chem. Lett.* 12:4286–4291. https://doi.org/10.1021/acs.jpclett.1c00681.

34. Schackert, F. K., J. Biedermann, …, H. Sun. 2023. Mechanism of calcium permeation in a glutamate receptor ion channel. *J. Chem. Inf. Model.* 63:1293–1300.

35. Antonides, L. H., Q. W. Hurst, …, U. Zachariae. 2022. The SARS-CoV-2 envelope (E) protein forms a calcium- and voltage-activated calcium channel. Preprint at bioRxiv. https://doi.org/10.1101/2022.10.11.511775.

36. Ives, C. M., N. J. Thomson, and U. Zachariae. 2023. A co-operative knock-on mechanism underpins Ca^{2+}-selective cation permeation in TRPV channels. *J. Gen. Physiol.* 155:e202213226. https://doi.org/10.1085/jgp.202213226.

37. Liu, C., L. Xue, and C. Song. 2023. Calcium binding and permeation in TRPV channels: insights from molecular dynamics simulations. *J. Gen. Physiol.* 155:e202213261.

38. Bauer, P., B. Hess, and E. Lindahl. 2022. GROMACS 2022 Source Code. https://doi.org/10.5281/zenodo.6103835.

39. Huang, J., S. Rauscher, …, A. D. MacKerell. 2017. CHARMM36m: An improved force field for folded and intrinsically disordered proteins. *Nat. Methods.* 14:71–73. https://doi.org/10.1038/nmeth.4067.

40. Jorgensen, W. L., J. Chandrasekhar, …, M. L. Klein. 1983. Comparison of simple potential functions for simulating liquid water. *J. Chem. Phys.* 79:926–935. https://doi.org/10.1063/1.445869.

41. Lee, J., X. Cheng, …, W. Im. 2016. CHARMM-GUI Input Generator for NAMD, GROMACS, AMBER, OpenMM, and CHARMM/OpenMM Simulations Using the CHARMM36 Additive Force Field. *J. Chem. Theor. Comput.* 12:405–413. https://doi.org/10.1021/acs.jctc.5b00935.

42. Evans, D. J., and B. L. Holian. 1985. The Nose-Hoover thermostat. *J. Chem. Phys.* 83:4069–4074. https://doi.org/10.1063/1.449071.

43. Parrinello, M., and A. Rahman. 1981. Polymorphic transitions in single crystals: A new molecular dynamics method. *J. Appl. Phys.* 52:7182–7190. https://doi.org/10.1063/1.328693.

44. Darden, T., D. York, and L. Pedersen. 1993. Particle mesh Ewald: An N·log(N) method for Ewald sums in large systems. *J. Chem. Phys.* 98:10089–10092. https://doi.org/10.1063/1.464397.

45. Hess, B., H. Bekker, …, J. G. E. M. Fraaije. 1997. LINCS: A Linear Constraint Solver for molecular simulations. *J. Comput. Chem.* 18:1463–1472. https://doi.org/10.1002/(sici)1096-987x(199709)18:12<1463::aid-jcc4>3.0.co;2-h.

46. Kutzner, C., H. Grubmüller, …, U. Zachariae. 2011. Computational Electrophysiology: The Molecular Dynamics of Ion Channel Permeation and Selectivity in Atomistic Detail. *Biophys. J.* 101:809–817. https://doi.org/10.1016/j.bpj.2011.06.010.

47. Kutzner, C., D. A. Köpfer, …, U. Zachariae. 2016. Insights into the function of ion channels by computational electrophysiology simulations. *Biochim. Biophys. Acta.* 1858:1741–1752. https://doi.org/10.1016/j.bbamem.2016.02.006.

48. Aksimentiev, A., and K. Schulten. 2005. Imaging α-Hemolysin with Molecular Dynamics: Ionic Conductance, Osmotic Permeability, and the Electrostatic Potential Map. *Biophys. J.* 88:3745–3761. https://doi.org/10.1529/biophysj.104.058727.

49. Hub, J. S., B. L. De Groot, and D. Van Der Spoel. 2010. g_wham, A Free Weighted Histogram Analysis Implementation Including Robust Error and Autocorrelation Estimates. *J. Chem. Theor. Comput.* 6:3713–3720.

50. Millman, K. J., and M. Aivazis. 2011. Python for Scientists and Engineers. https://doi.org/10.1109/mcse.2011.36.

51. Oliphant, T. E. 2007. Python for scientific computing. *Comput. Sci. Eng.* 9:10–20. https://doi.org/10.1109/mcse.2007.58.

52. Pérez, F., and B. E. Granger. 2007. IPython: A system for interactive scientific computing. *Comput. Sci. Eng.* 9:21–29. https://doi.org/10.1109/mcse.2007.53.

53. Van Der Walt, S., S. C. Colbert, and G. Varoquaux. 2011. The NumPy array: A structure for efficient numerical computation. *Comput. Sci. Eng.* 13:22–30. https://doi.org/10.1109/mcse.2011.37.

54. Gowers, R., M. Linke, …, O. Beckstein. 2016. MDAnalysis: A Python Package for the Rapid Analysis of Molecular Dynamics Simulations. *In* Proceedings of the 15th Python in Science Conference, pp. 98–105. https://doi.org/10.25080/majora-629e541a-00e.

55. Michaud-Agrawal, N., E. J. Denning, …, O. Beckstein. 2011. MDAnalysis: A toolkit for the analysis of molecular dynamics simulations. *J. Comput. Chem.* 32:2319–2327. https://doi.org/10.1002/jcc.21787.

56. Klesse, G., M. S. P. P. Sansom, …, G. Klesse. 2019. CHAP: A Versatile Tool for the Structural and Functional Annotation of Ion Channel Pores. *J. Mol. Biol.* 431:3353–3365. https://doi.org/10.1016/j.jmb.2019.06.003.

57. Hunter, J. D. 2007. Matplotlib: A 2D graphics environment. *Comput. Sci. Eng.* 9:90–95. https://doi.org/10.1109/mcse.2007.55.

58. Waskom, M., O. Botvinnik…, 2018. mwaskom/seaborn: v0.9.0. https://doi.org/10.5281/zenodo.1313201.

59. Thomson, N. J., O. N. Vickery, …, U. Zachariae. 2021. Ion-water coupling controls class A GPCR signal transduction pathways. Preprint at bioRxiv. https://doi.org/10.1101/2020.08.28.271510.

60. Vögele, M., N. J. Thomson, …, R. O. Dror. 2022. Systematic Analysis of Biomolecular Conformational Ensembles with PENSA. Preprint at arXiv. https://doi.org/10.48550/arXiv.2212.02714.

61. McClendon, C. L., G. Friedland, …, M. P. Jacobson. 2009. Quantifying correlations between allosteric sites in thermodynamic ensembles. *J. Chem. Theor. Comput.* 5:2486–2502. https://doi.org/10.1021/ct9001812.

62. Pethel, S., and D. Hahs. 2014. Exact Test of Independence Using Mutual Information. *Entropy.* 16:2839–2849. https://doi.org/10.3390/e16052839.

63. Prawitt, D., M. K. Monteilh-Zoller, …, R. Penner. 2003. TRPM5 is a transient Ca2+-activated cation channel responding to rapid changes in [Ca2+] i. *Proc. Natl. Acad. Sci. USA.* 100:15166–15171.

64. Biedermann, J., S. Braunbeck, …, H. Sun. 2021. Nonselective cation permeation in an AMPA-type glutamate receptor. *Proc. Natl. Acad. Sci. USA.* 118:e2012843118.

65. Milenkovic, S., I. V. Bodrenko, …, M. Ceccarelli. 2021. The key role of the central cavity in sodium transport through ligand-gated two-pore channels. *Phys. Chem. Chem. Phys.* 23:18461–18474.

66. Bucher, D., L. Guidoni, …, U. Rothlisberger. 2010. Coordination numbers of K+ and Na+ ions inside the selectivity filter of the KcsA potassium channel: Insights from first principles molecular dynamics. *Biophys. J.* 98:L47–L49.

67. Zhang, J., D. Song, …, C. Shi. 2023. Fluoride permeation mechanism of the Fluc channel in liposomes revealed by solid-state NMR. *Sci. Adv.* 9:eadg9709.

68. Noskov, S. Y., and B. Roux. 2007. Importance of hydration and dynamics on the selectivity of the KcsA and NaK channels. *J. Gen. Physiol.* 129:135–143.

69. Marcus, Y. 1991. Thermodynamics of solvation of ions. Part 5.—Gibbs free energy of hydration at 298.15 K. *J. Chem. Soc., Faraday Trans.* 87:2995–2999.

70. Doyle, D. A., J. Morais Cabral, …, R. MacKinnon. 1998. The structure of the potassium channel: molecular basis of K+ conduction and selectivity. *Science.* 280:69–77.

71. Aryal, P., F. Abd-Wahab, …, S. J. Tucker. 2014. A hydrophobic barrier deep within the inner pore of the TWIK-1 K2P potassium channel. *Nat. Commun.* 5:4377.

72. Jia, Z., M. Yazdani, …, J. Chen. 2018. Hydrophobic gating in BK channels. *Nat. Commun.* 9:3408.

73. Nordquist, E. B., Z. Jia, and J. Chen. 2023. Inner pore hydration free energy controls the activation of big potassium channels. *Biophys. J.* 122:1158–1167.

74. Sievers, F., A. Wilm, …, D. G. Higgins. 2011. Fast, scalable generation of high-quality protein multiple sequence alignments using Clustal Omega. *Mol. Syst. Biol.* 7:539.

75. Yariv, B., E. Yariv, …, N. Ben-Tal. 2023. Using evolutionary data to make sense of macromolecules with a “face-lifted” ConSurf. *Protein Sci.* 32:e4582. https://doi.org/10.1002/pro.4582.

76. Consortium, T. U. 2022. UniProt: the Universal Protein Knowledgebase in 2023. *Nucleic Acids Res.* 51:D523–D531. https://doi.org/10.1093/nar/gkac1052.

77. DeLano, W. L. 2002. Pymol: An open-source molecular graphics tool. *CCP4 Newsletter on protein crystallography.* 40:82–92. http://www.ccp4.ac.uk/newsletters/newsletter40/11_pymol.pdf.

78. Born, M. 1920. Volumen und hydratationswärme der ionen. *Z. Phys.* 1:45–48.

79. Sakipov, S., A. I. Sobolevsky, and M. G. Kurnikova. 2018. Ion permeation mechanism in epithelial calcium channel TRVP6. *Sci. Rep.* 8:5715.

80. Hille, B. 2001. Ion Channels of Excitable Membranes, 3rd ed. Sinauer, Sunderland, MA.

81. Zhou, Y., J. H. Morais-Cabral, …, R. MacKinnon. 2001. Chemistry of ion coordination and hydration revealed by a K+ channel–Fab complex at 2.0 Å resolution. *Nature.* 414:43–48.

82. Derebe, M. G., D. B. Sauer, …, Y. Jiang. 2011. Tuning the ion selectivity of tetrameric cation channels by changing the number of ion binding sites. *Proc. Natl. Acad. Sci. USA.* 108:598–602.

83. Köpfer, D. A., C. Song, …, B. L. De Groot. 2014. Ion permeation in K^+ channels occurs by direct Coulomb knock-on. *Science.* 346:352–355. https://doi.org/10.1126/science.1254840.

84. Duan, J., Z. Li, …, D. E. Clapham. 2018. Structure of full-length human TRPM4. *Proc. Natl. Acad. Sci. USA.* 115:2377–2382.

85. Guo, J., J. She, …, Y. Jiang. 2017. Structures of the calcium-activated, non-selective cation channel TRPM4. *Nature.* 552:205–209.

86. Vögele, M., N. J. Thomson, …, J. McAvity. 2021. drorlab/pensa: PENSA 0.2.7. https://doi.org/10.5281/zenodo.5522808.

Biophysical Journal, Volume *123*

Supplemental information

A hydrophobic funnel governs monovalent cation selectivity in the ion channel TRPM5

Callum M. Ives, Alp Tegin Şahin, Neil J. Thomson, and Ulrich Zachariae

Supplementary Information

Summary of MD simulations used within this study

Table S1. Summary of CompEL simulation details of the TRPM5 channel. All simulations were conducted in a di-cationic solution of 75 mM NaCl and 75 mM $CaCl_2$. In all simulations, the Ca^{2+} cations occupying the Ca_{TMD} were modelled, and remained bound for the duration of the simulations.

**Protein**	TRPM5				
**Structure**	7MBS<br>(698-1020)				
**Force field**	CHARMM36m				
**Water**	TIP3P				
In silico electrophysiology methodology	CompEL<br>(anti-parallel with a 9:1 concentration gradient)				
**Ion**	75 mM NaCl + 75mm $CaCl_2$<br>266 Na^+ (CHARMM36m)<br>274 Ca^{2+} (Zhang et al.)<br>814 Cl^- (CHARMM36m)				
**Independent simulations**	3	3	3	3	3
**Total simulation time (μs)**	1.5	1.5	1.5	1.5	0.45
**Total aggregated simulation time (μs)**	3	3	3	3	0.9
**Ionic ratios between compartments (Extracellular : Intracellular)**	239 : 27 Na^+<br>239 : 35 Ca^{2+}<br>681 : 133 Cl^-	239 : 27 Na^+<br>239 : 35 Ca^{2+}<br>680 : 134 Cl^-	239 : 27 Na^+<br>239 : 35 Ca^{2+}<br>677 : 137 Cl^-	239 : 27 Na^+<br>239 : 35 Ca^{2+}<br>673 : 141 Cl^-	239 : 27 Na^+<br>239 : 35 Ca^{2+}<br>684 : 130 Cl^-
**Estimated voltage (mV)**	-50	-130	-380	-610	0
**Permeation events**	15 Na^+<br>0 Ca^{2+}<br>0 Cl^-	18 Na^+<br>0 Ca^{2+}<br>0 Cl^-	32 Na^+<br>19 Ca^{2+}<br>1 Cl^-	115 Na^+<br>168 Ca^{2+}<br>6 Cl^-	0 Na^+<br>0 Ca^{2+}<br>0 Cl^-
**Total number of permeation events**	15	18	52	289	0

Table S2. Summary of external applied field simulation details of the TRPM5 channel. All simulations were conducted in a mono-cationic solution of either 150 mM NaCl, 150 mM KCl, or 150 mM $CaCl_2$. In all simulations, the Ca^{2+} cations occupying the Ca_{TMD} were modelled, and remained bound for the duration of the simulations.

**Protein**	TRPM5		
**Structure**	7MBS<br>(698-1020)		
**Force field**	CHARMM36m		
**Water**	TIP3P		
In silico electrophysiology methodology	External applied field		
**Ion**	150 mM NaCl<br>267 Na^+ (CHARMM36m)<br>275 Cl^- (CHARMM36m)<br>4 Ca^{2+} (CHARMM36m)	150 mM KCl<br>267 K+ (CHARMM36m)<br>275 Cl^- (CHARMM36m)<br>4 Ca^{2+} (CHARMM36m)	150 mM $CaCl_2$<br>271 Ca^{2+} (Zhang *et al*.)<br>542 Cl^- (CHARMM36m)
**Independent simulations**	3	3	3
**Total simulation time (μs)**	0.75	0.75	0.75
**Estimated voltage (mV)**	-340	-340	-340
**Permeation events**	83 Na^+<br>0 Cl^-	34 K+<br>0 Cl^-	54 Ca^{2+}<br>0 Cl^-
**Total number of permeation events**	83	34	54

**Protein**	TRPM5			
**Structure**	7MBS<br>(698-1020)			
**Force field**	CHARMM36m			
**Water**	TIP3P			
In silico electrophysiology methodology	External applied field			
**Ion**	150 mM NaCl<br>267 Na^+ (CHARMM36m)<br>275 Cl^- (CHARMM36m)<br>4 Ca^{2+} (CHARMM36m)	150 mM $CaCl_2$<br>271 Ca^{2+} (Zhang et al.)<br>542 Cl^- (CHARMM36m)	150 mM NaCl<br>267 Na^+ (CHARMM36m)<br>275 Cl^- (CHARMM36m)<br>4 Ca^{2+} (CHARMM36m)	150 mM $CaCl_2$<br>271 Ca^{2+} (Zhang et al.)<br>542 Cl^- (CHARMM36m)
**Independent simulations**	3	3	3	3
**Total simulation time (μs)**	0.75	0.75	0.75	0.75
**Estimated voltage (mV)**	-50	-50	-200	-200
**Permeation events**	4 Na^+<br>0 Cl^-	0 Ca^{2+}<br>0 Cl^-	15 Na^+<br>0 Cl^-	4 Ca^{2+}<br>0 Cl^-
**Total number of permeation events**	4	0	15	4

Table S3. Summary of external applied field simulation details of the TRPM5 F904T channel. All simulations were conducted in a mono-cationic solution of either 150 mM NaCl, or 150 mM $CaCl_2$. In all simulations, the Ca^{2+} cations occupying the Ca_{TMD} were modelled, and remained bound for the duration of the simulations.

**Protein**	TRPM5 F904T			
**Structure**	7MBS<br>(698-1020)			
**Force field**	CHARMM36m			
**Water**	TIP3P			
**In silico electrophysiology methodology**	External applied field			
**Ion**	150 mM NaCl<br>267 Na+ (CHARMM36m)<br>275 Cl- (CHARMM36m)<br>4 Ca2+ (CHARMM36m)	150 mM CaCl2<br>271 Ca2+ (Zhang et al.)<br>542 Cl- (CHARMM36m)	150 mM NaCl<br>267 Na+ (CHARMM36m)<br>275 Cl- (CHARMM36m)<br>4 Ca2+ (CHARMM36m)	150 mM CaCl2<br>271 Ca2+ (Zhang et al.)<br>542 Cl- (CHARMM36m)
**Independent simulations**	3	3	3	3
**Total simulation time (μs)**	0.75	0.75	0.75	0.75
**Estimated voltage (mV)**	-130	-130	-200	-200
**Permeation events**	27 Na+<br>0 Cl-	13 Ca2+<br>0 Cl-	88 Na+<br>0 Cl-	28 Ca2+<br>0 Cl-
**Total number of permeation events**	27	13	88	28

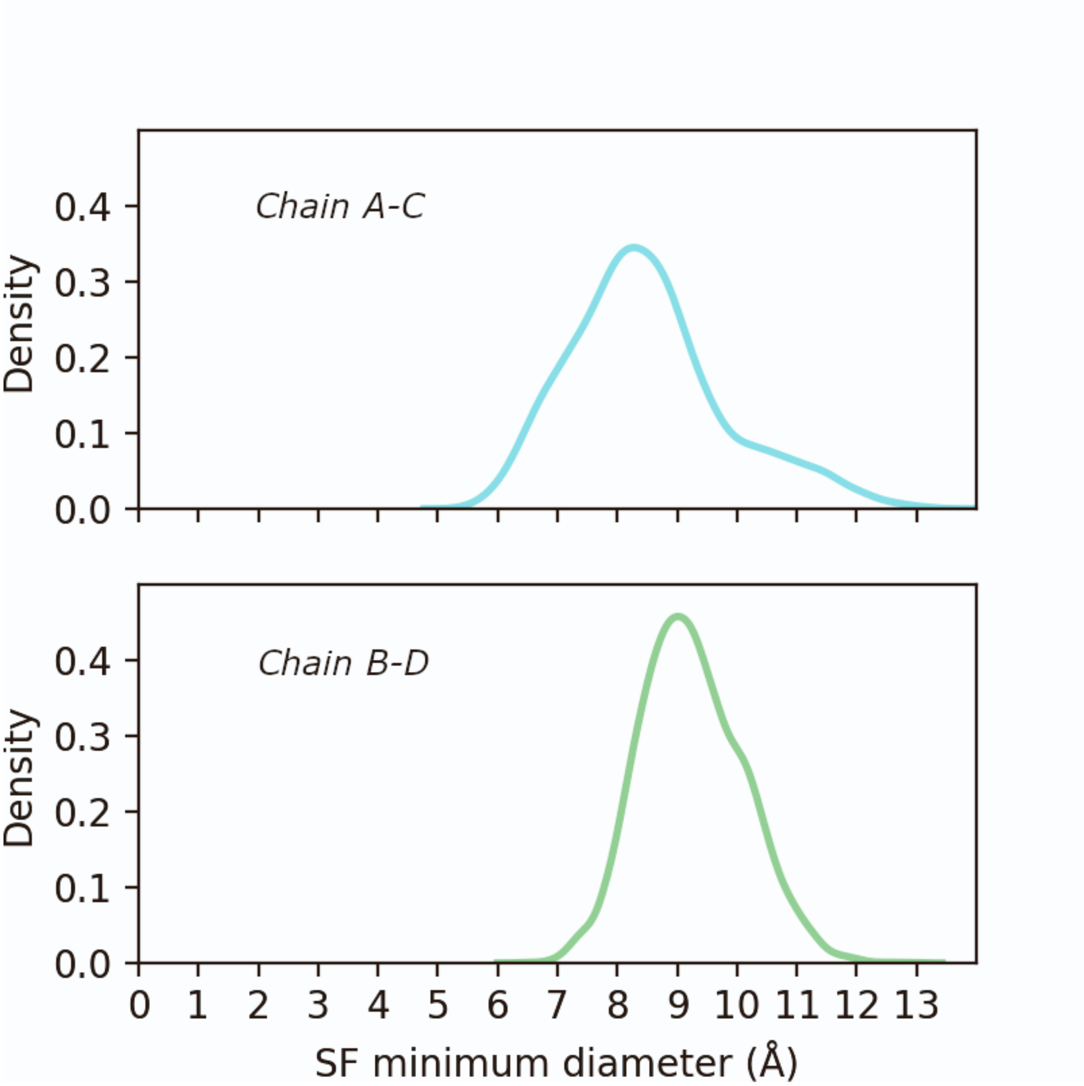

Figure S1. Minimum diameter of the TRPM5 selectivity filter measured at the SF constriction formed by residues G905 (carbonyl groups). The top panel shows the distance between the residues of chains A and C, the lower panel between chains B and D.

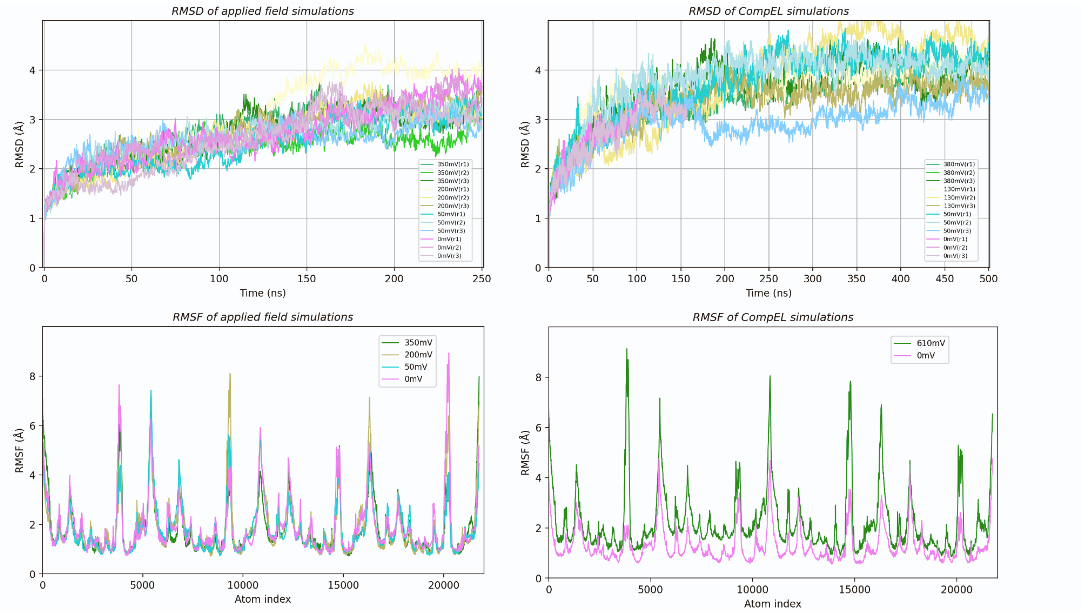

Figure S2. Root-mean-square deviation (RMSD) and root-mean-square fluctuation (RMSF) of the channel in the simulations. Note that the CompEL simulations at 0 mV are shorter than those at a higher voltage.

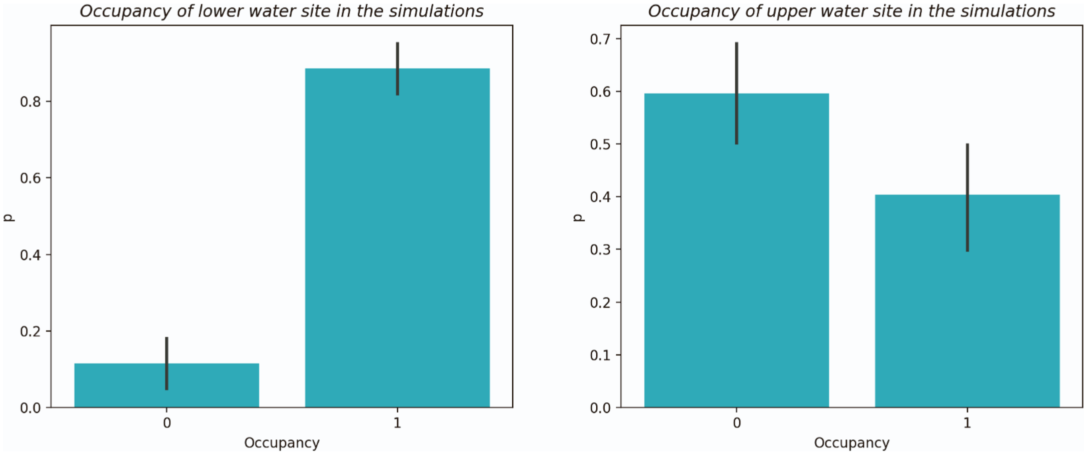

Figure S3. Occupancy of the water sites near Q906 in the SF identified in the cryo-EM structures.

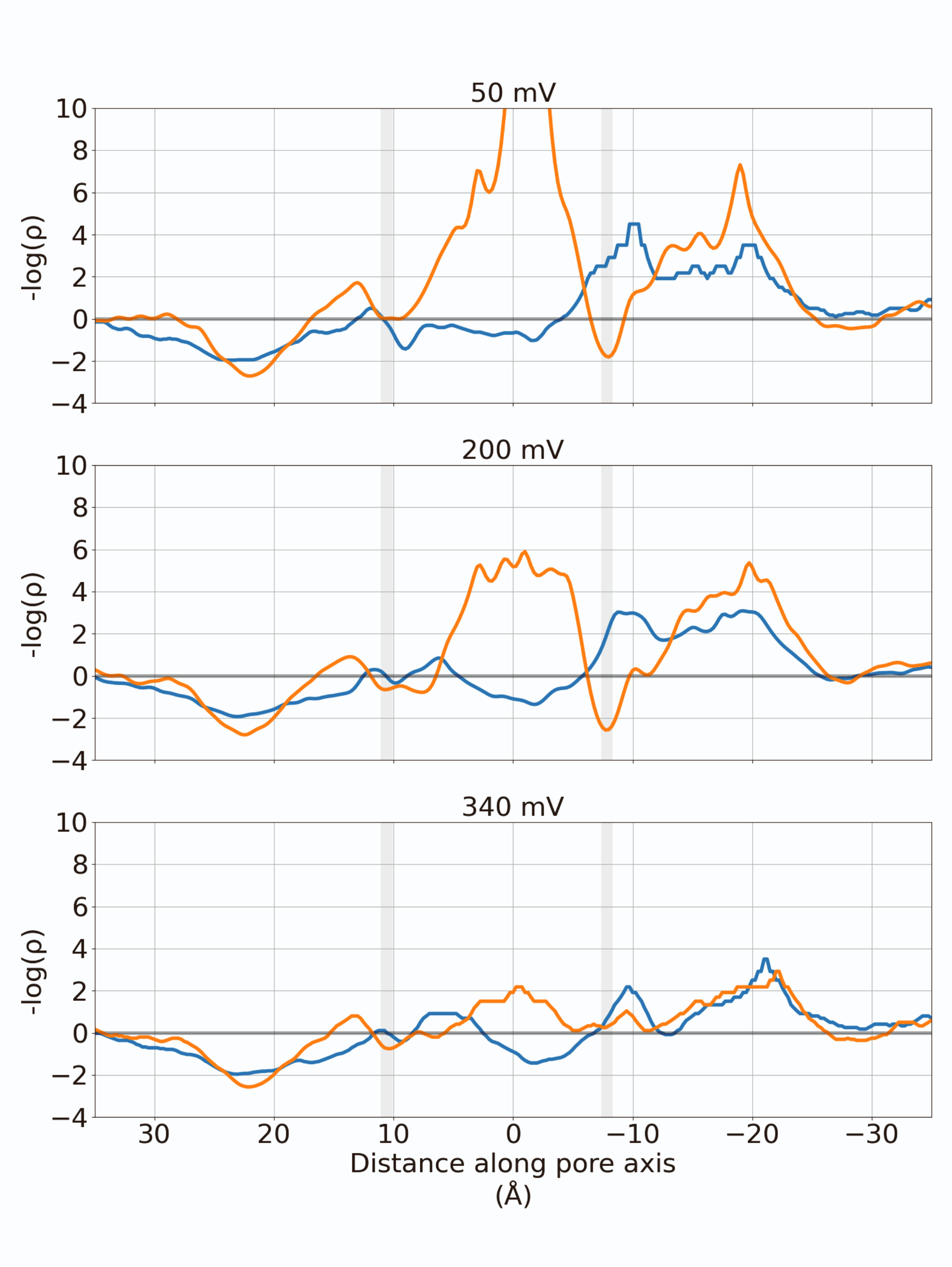

Figure S4. Negative logarithmic density profiles of permeating cations along the pore of the TRPM5 at different voltages. These simulations were performed in a mono-cationic solution, with an external applied electric field used to produce transmembrane voltages of ~ -50 mV (*top*), ~ -200 mV (*centre*), and ~ -340 mV (*bottom*). The logarithmic ion densities represent quasi-free energies (with a nominal unit of kT). The location of the pore constrictions formed by Q906 (*upper*) and I966 (*lower*) are represented as gray regions. Both plots have been smoothed using a Gaussian filter with a sigma value of 2.

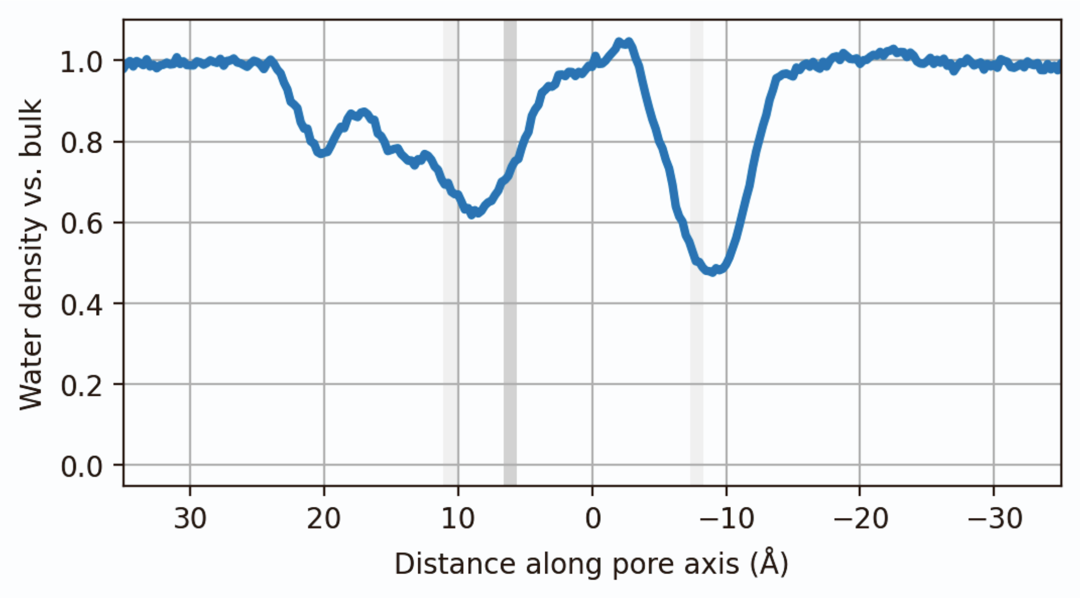

Figure S5. Water density along the pore axis of TRPM5 compared to bulk solution. Functionally important residue locations in the pore are shown as gray bars.
